# Iron-related gene mutations driving global *Mycobacterium tuberculosis* transmission revealed by whole-genome sequencing

**DOI:** 10.1186/s12864-024-10152-1

**Published:** 2024-03-06

**Authors:** Yameng Li, Yifan Li, Yao Liu, Xianglong Kong, Ningning Tao, Yawei Hou, Tingting Wang, Qilin Han, Yuzhen Zhang, Fei Long, Huaichen Li

**Affiliations:** 1 Clinical Department of Integrated Traditional Chinese and Western Medicine , The First Clinical Medical College of Shandong University of Traditional Chinese Medicine, 250014 Jinan, Shandong People’s Republic of China; 2 Department of Pulmonary and Critical Care Medicine, The Third Affiliated Hospital of Shandong First Medical University (Affiliated Hospital of Shandong Academy of Medical Sciences), 250031 Jinan, Shandong People’s Republic of China; 3 Department of Pulmonary and Critical Care Medicine, Shandong Provincial Hospital Affiliated to Shandong University, Shandong Provincial Hospital Affiliated to Shandong First Medical University, 250021 Jinan, Shandong People’s Republic of China; 4 Artificial Intelligence Institute, Qilu University of Technology (Shandong Academy of Sciences), 250011 Jinan, Shandong People’s Republic of China; 5 Institute of Chinese Medical Literature and Culture of Shandong University of Traditional Chinese Medicine, 250355 Jinan, Shandong People’s Republic of China; 6 Shandong First Medical University & Shandong Academy of Medical Sciences, 250117 Jinan, Shandong People’s Republic of China

**Keywords:** Iron-related gene mutations, *Mycobacterium tuberculosis*, Transmission, Whole-genome sequencing

## Abstract

**Background:**

Iron plays a crucial role in the growth of *Mycobacterium tuberculosis* (*M. tuberculosis*). However, the precise regulatory mechanism governing this system requires further elucidation. Additionally, limited studies have examined the impact of gene mutations related to iron on the transmission of *M. tuberculosis* globally. This research aims to investigate the correlation between mutations in iron-related genes and the worldwide transmission of *M. tuberculosis*.

**Results:**

A total of 13,532 isolates of *M. tuberculosis* were included in this study. Among them, 6,104 (45.11%) were identified as genomic clustered isolates, while 8,395 (62.04%) were classified as genomic clade isolates. Our results showed that a total of 12 single nucleotide polymorphisms (SNPs) showed a positive correlation with clustering, such as *Rv1469* (*ctpD*, C758T), *Rv3703c* (*etgB*, G1122T), and *Rv3743c* (*ctpJ*, G676C). Additionally, seven SNPs, including *Rv0104* (T167G, T478G), *Rv0211* (*pckA*, A302C), *Rv0283* (*eccB3*, C423T), *Rv1436* (*gap*, G654T), *ctpD* C758T, and *etgB* C578A, demonstrated a positive correlation with transmission clades across different countries. Notably, our findings highlighted the positive association of *Rv0104* T167G, *pckA* A302C, *eccB3* C423T, *ctpD* C758T, and *etgB* C578A with transmission clades across diverse regions. Furthermore, our analysis identified 78 SNPs that exhibited significant associations with clade size.

**Conclusions:**

Our study reveals the link between iron-related gene SNPs and *M. tuberculosis* transmission, offering insights into crucial factors influencing the pathogenicity of the disease. This research holds promise for targeted strategies in prevention and treatment, advancing research and interventions in this field.

**Supplementary Information:**

The online version contains supplementary material available at 10.1186/s12864-024-10152-1.

## Background

Tuberculosis (TB) is an airborne infectious disease caused by *Mycobacterium tuberculosis* (*M. tuberculosis*) and is the leading cause of death worldwide among infectious diseases. Despite great progress over the past decades, TB remains a major global health problem. In 2022, TB remained the second leading cause of death from a single infectious agent, after coronavirus disease (COVID-19), and caused almost twice as many deaths as HIV/AIDS [1]. Globally, there were 7.5 million newly diagnosed cases of TB reported in 2022. Additionally, the total number of deaths attributed to TB, including those among individuals with HIV, reached 1.30 million during the same year [1]. Despite the immense global burden of tuberculosis, our understanding of the factors influencing its transmission remains limited. Therefore, gaining a deeper insight into the mechanisms underlying the transmission of *M. tuberculosis* is imperative in order to inform and guide effective strategies for tuberculosis control, ultimately leading to a reduction in the societal burden imposed by this disease.

Iron holds paramount importance as an indispensable element for nearly all living organisms due to its involvement in a vast array of metabolic processes, encompassing oxygen transportation, DNA synthesis, and electron conveyance [2]. In the context of *M. tuberculosis*, iron emerges as an essential catalyst for growth. The significance of iron in the growth and metabolism of bacteria is elucidated through its acquisition from host reservoirs like transferrin, lactoferrin, and ferritin, followed by subsequent assimilation and utilization within the bacterial framework. Crucial constituents participating in the procurement of iron (in the form of ferric ion) and its preliminary transference into the mycobacterium cell encompass extracellular iron-binding agents, known as siderophores. In pathogenic mycobacteria, carboxymycobactins fulfill this role, while exochelins perform analogous functions in saprophytic mycobacteria [3, 4]. Upon successful acquisition, the next imperative step entails transporting iron across the mycobacterium cell membrane. *M. tuberculosis* employs specialized systems to facilitate this process. Subsequently, inside the mycobacterium cell, iron finds employment in diverse metabolic pathways, functioning as a pivotal cofactor for enzymes engaged in critical processes such as DNA synthesis, respiration, and energy production [3, 5]. Furthermore, iron plays a crucial role in regulating gene expression and maintaining redox homeostasis [6]. *M. tuberculosis* exploits iron to disrupt host immune responses, thereby enhancing its survival and dissemination. In summary, iron contributes to the establishment and survival of *M. tuberculosis* within the host. By utilizing the iron resources, the bacterium can better adapt to the host environment and increase its transmission capacity. Iron plays a key role in the growth, pathogenicity, immune evasion, and host adaptation of *M. tuberculosis*. However, the specific regulatory mechanisms of iron-related genes involved in the dissemination of *M. tuberculosis* remain unclear. Further research is needed to uncover these mechanisms, providing insights into the pathogenesis of tuberculosis and facilitating the development of more effective treatment strategies.

Whole-genome sequencing (WGS) is progressively being used to investigate the transmission dynamics of *M. tuberculosis*. In this study, we employed WGS to analyze the impact of mutations in iron-related genes on the global transmission of *M. tuberculosis*. Specifically, the genome cluster and clade were used to represent the transmission of *M. tuberculosis*.

## Method

### Sample Collection

Between 2011 and 2018, a total of 1,550 culture-positive cases of *M. tuberculosis* were collected from two medical institutions in China, namely the Shandong Public Health Clinical Research Center (SPHCC) and the Weifang Respiratory Clinical Hospital (WRCH). The study did not include cases with positive culture of *M. tuberculosis* that were previously evaluated and subsequently treated.

### DNA extraction and sequencing

A total of 1447 isolates were included in this study, and genomic DNA was extracted from these isolates using the Cetyltrimethylammonium Bromide (CTAB) method. Prior to analysis, quality control (QC) procedures were conducted on the extracted DNA. However, 103 isolates of *M. tuberculosis* were excluded from further analysis due to issues related to improper handling during DNA extraction and poor quality of the extracted DNA. For the remaining isolates, their genomes were sequenced utilizing the Illumina HiSeq 4000 system. The resulting sequence data were then deposited in the National Center for Biotechnology Information (NCBI) under the BioProject PRJNA1002108. In addition to the aforementioned isolates, this study also included a larger dataset consisting of 13,267 isolates of *M. tuberculosis* collected from 52 countries and 18 regions worldwide, as reported in previous studies [7–15]. To accurately map the reference genome of the standard isolate *M. tuberculosis* H37Rv, we employed the BWA-MEM (version 0.7.17-r1188). Our analysis focused solely on samples with a coverage rate of 98% or higher and a minimum depth of at least 20× [16]. In summary, a total of 13,532 genomes were analyzed in this study, please refer to Additional file 1: Tables S14-S15 for the specific sample numbers.

### Single nucleotide polymorphism (SNP) analysis

We performed variant calling using Samclip (version 0.4.0) and SAMtools (version 1.15). Following variant calling, we applied additional filtering steps to refine the resulting variants. This involved utilizing Free Bayes (version 1.3.2) and Bcftools (version 1.15.1) for further variant filtering. To ensure the accuracy of our analysis, we excluded SNPs located within repeat regions. This included polymorphic GC-rich sequences found in PE/PPE genes, direct repeat SNPs, and repeat bases identified through the use of Tandem Repeat Finder (version 4.09) and RepeatMask (version 4.1.2-P1) [17, 18]. Finally, SNP annotation was conducted using SnpEff v 4.1 l. The resulting output was obtained by utilizing the Python programming language [19].

### Phylogenetic analysis

According to Coll et al [20] (Additional file 2: Tables S14-S15), the isolates in this study were classified into different lineages. To construct the maximum likelihood phylogenetic tree, we utilized the IQ-TREE software package (version 1.6.12). The JC nucleotide substitution model and gamma model of rate heterogeneity were used, with 100 bootstrap replicates included for statistical support [21]. During the analysis, *Mycobacterium canettii CIPT140010059* was identified as an outlier and was treated accordingly. The resulting phylogenetic tree was visualized using iTOL (https://itol.embl.de/) for better representation and interpretation.

### Propagation analysis

We employed cluster and clade analysis to investigate the impact of mutations in iron-related genes on the transmission of *M. tuberculosis* [22]. Expanding upon previous studies [23], clustering techniques were utilized to define transmitted clusters, using a threshold of less than 12 SNPs. Additionally, clade analysis was conducted to identify transmission clades, with a threshold of less than 25 SNPs. To further categorize the transmission clades, we adopted a classification system used by scholars. The clades were classified into three groups based on size: large (above the 75th percentile), medium (between the 25th and 75th percentiles), and small (below the 25th percentile) [24]. For a comprehensive analysis of the global distribution patterns and transmission dynamics of *M. tuberculosis* isolates, we classified them into cross-country and within-country clades. Cross-country clades consist of isolates from two or more different countries. Furthermore, based on geographic location, the *M. tuberculosis* isolates were classified into cross-regional and within-regional clades using the United Nations standard regions (UN M.49). Cross-regional clades include isolates from two or more different regions.

### Acquisition of iron-related genes

In our study, we obtained genes related to iron in *M. tuberculosis* from the NCBI database, which were previously discovered by scholars. These genes encompass various aspects such as iron uptake transporters, iron storage proteins, iron-regulated transcription factors, and enzymes involved in iron-dependent processes. A total of 59 iron-related genes were retrieved from the NCBI database. Python was utilized to detect mutations in genes associated with iron (Additional file 1: Table S16).

### Modeling and statistical analysis

The data were presented as percentages. Positions with mutation frequency below 0.01 in the iron-related genes were excluded from the analysis [25]. For statistical analysis, we employed generalized linear mixed models in the R statistical language (R 4.2.3). To further analyze the data, random forest and gradient boosting decision tree algorithms were implemented using Python 3.7.4 with the Scikit-learn library (Python Software Foundation, USA; Packt Publishing, UK). The dataset was randomly divided into a training set and a test set in a 7:3 ratio. In order to assess the impact of mutations in iron-related genes on clade size, Spearman’s rank correlation analysis was performed using R version 4.2.3. Confounding factors such as lineage and geographical location were taken into account during all analyses. All statistical analyses were conducted using SPSS 26.0. Two-tailed tests were used, and statistical significance was defined as a *P*-value below 0.05.

## Results

### Sample description

**Fig. 1:**
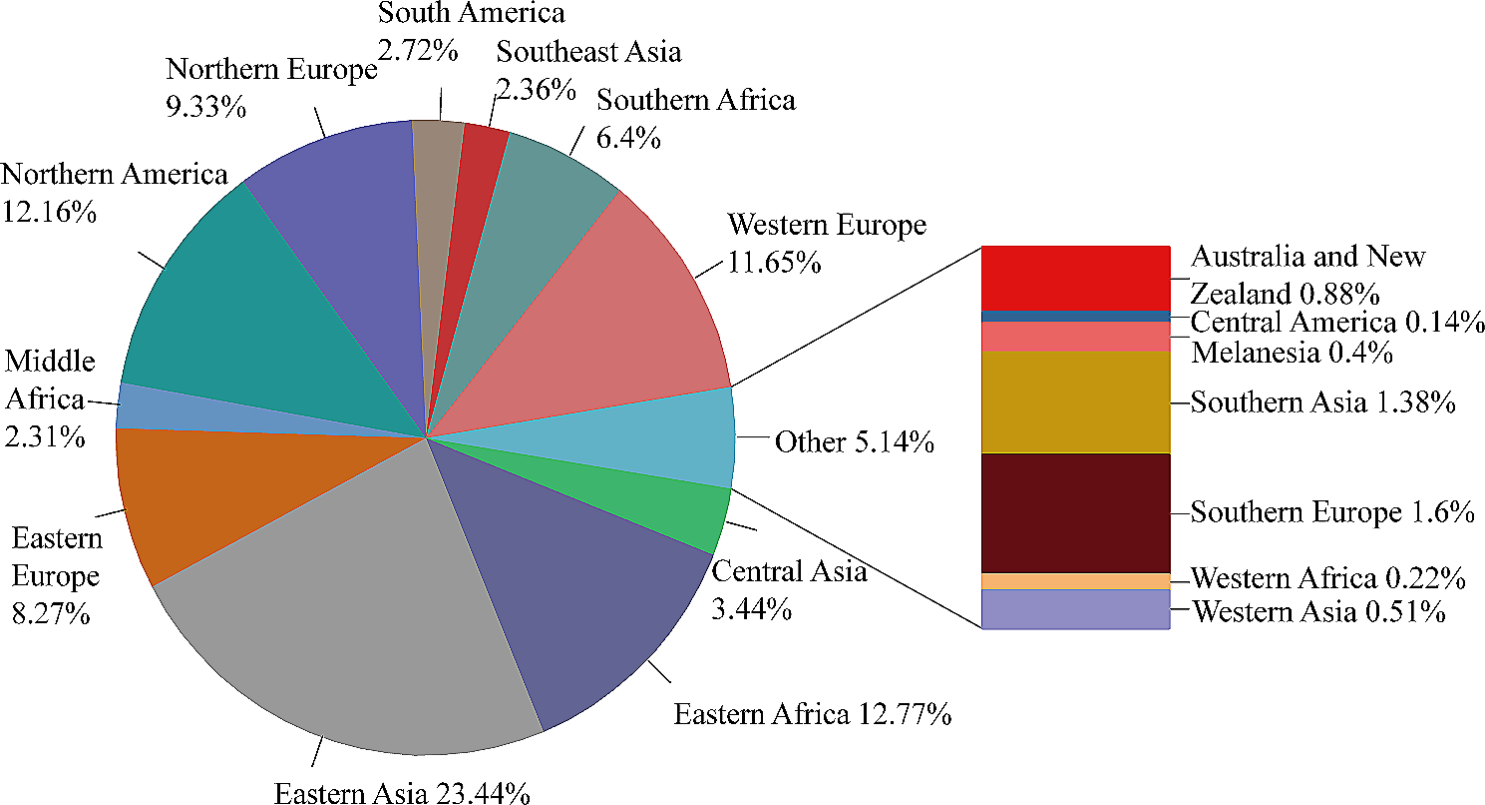
Distribution of *Mycobacterium tuberculosis* in various regions

**Fig. 2:**
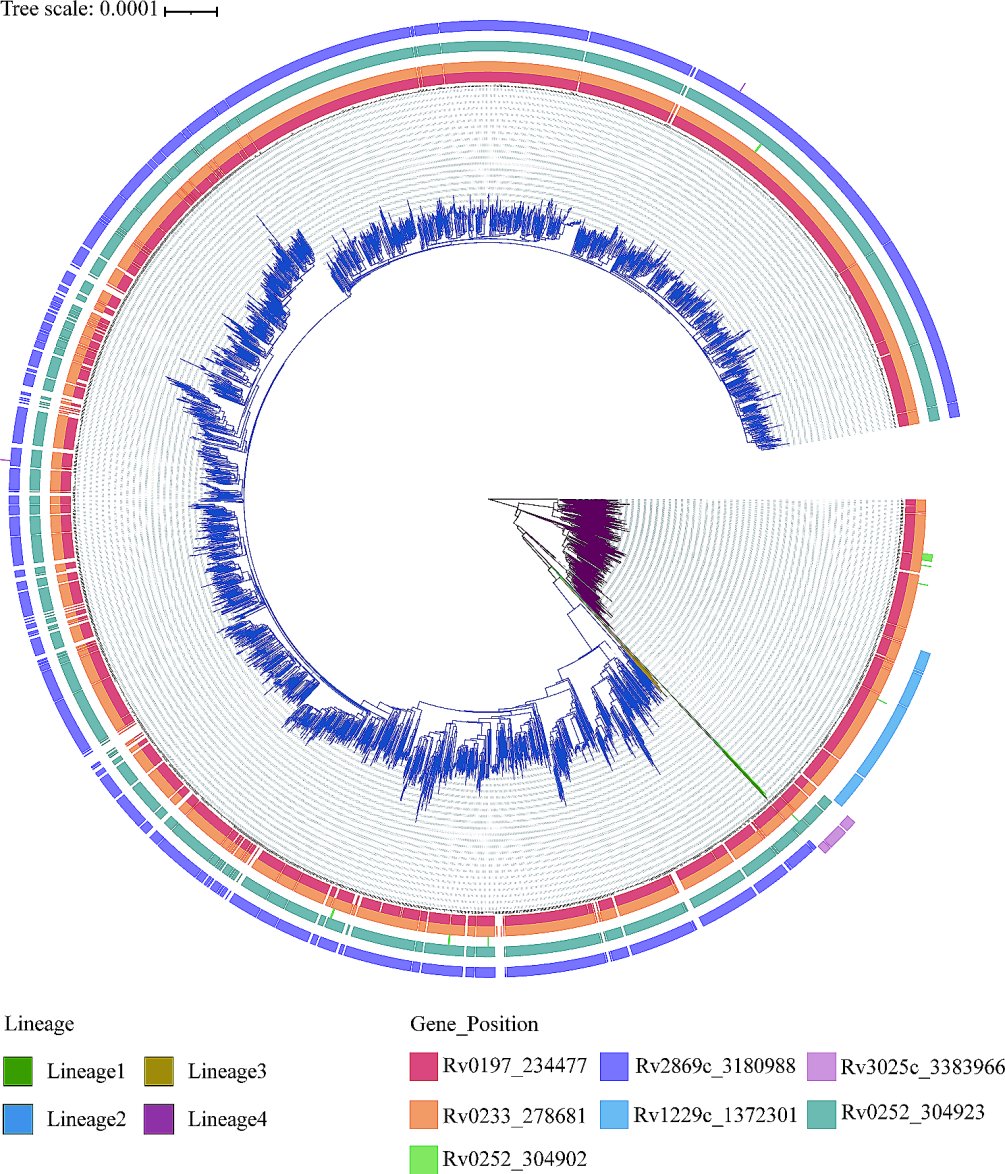
Phylogenetic tree for the *Mycobacterium tuberculosis* isolates from China

We included a total of 13,532 isolates of *M. tuberculosis* from around the world, with 1,445 isolates collected between 2011 and 2018 at the Shandong Public Health Clinical Research Center (SPHCC) and the Weifang Respiratory Clinical Hospital (WRCH). Among these isolates, the highest proportion was observed in Eastern Asia (*n* = 3,172, 23.44%), followed by Eastern Africa (*n* = 1,728, 12.77%) and Northern America (*n* = 1,646, 12.16%), as depicted in Fig. 1. Additionally, the majority of these isolates (*n* = 6,499, 48.03%) belonged to lineage 4, while (*n* = 5,135, 37.95%) belonged to lineage 2, aligning with our expectations. Isolates were divided into clusters based on < = 12 single nucleotide polymorphisms (SNPs). Accordingly, a total of 6,104 isolates clustered together, resulting in a clustering rate of 0.45. Within the lineage 4 group, 2,971 (45.71%) isolates formed clusters, while within the lineage 2 group, 2,131 (41.50%) isolates formed clusters. When applying a threshold of 25 SNPs for clades, a total of 8,395 isolates clade together, resulting in a clade rate of 0.62. The *M. tuberculosis* isolates were further grouped into 2,218 clades, with the number of isolates per clade ranging from 2 to 224 isolates. Within these clades, there were 177 cross-country clades, consisting of 2 to 4 countries, and 171 cross-regional clades, consisting of 2 to 4 regions, as shown in Table 1. The phylogenetic tree of *M. tuberculosis* isolates was constructed as described in Fig. 2.

**Table 1:** The characteristics of *Mycobacterium tuberculosis* isolates

Characteristic				Number of isolates (%)
**Lineage**	lineage1			851(6.29)
	lineage2			5135(37.95)
	lineage3			970(7.17)
	lineage4			6499(48.03)
	lineage5			38(0.28)
	lineage6			10(0.07)
	lineage7			29(0.21)
**12 SNPs**	Cluster			6104(45.11)
	No-cluster			7428(54.89)
	Lineage2	cluster		2131(41.50)
		no-cluster		3004(58.50)
	Lineage4	cluster		2971(45.71)
		no-cluster		3528(54.29)
**25 SNPs**	Clade			8395(62.04)
	No-Clade			5137(37.96)
	Cross-country		Yes	720(8.58)
			No	7675(91.42)
	Cross-regional		Yes	701(8.35)
			No	7694(91.65)
	Clades by size		small	2732(32.54)
			medium	3519(41.92)
			large	2144(25.54)

### Relationship between iron-related gene mutations and transmission clusters

After excluding sites with a mutation frequency below 0.01, we identified and included a total of 90 SNPs for further analysis. Subsequently, we conducted a comparative analysis between clustered and non-clustered isolates, examining the relationship between these 90 SNPs and the occurrence of clustering. The generalized linear mixed model (GLMM) revealed that 21 SNPs were statistically significant for clustering (*P* < 0.05) (Additional file 1: Table S1). Among these, eight nonsynonymous SNPs and five synonymous SNPs showed a positive correlation with transmission clusters in *M. tuberculosis* isolates. The specific SNPs included *Rv0197* (G344T, T2247G), *Rv0252* (*nirB*, C2037T, A2058G), *Rv0338c* C2478T, *Rv1229c* (*mrp*, C649G), *Rv1436* (*gap*, G654T), *Rv1469* (ctpD, C758T), *Rv2869c* (*rip*, C957T, G775T), *Rv3703c* (*etgB*, G1122T), *Rv3728* (C2392T), and *Rv3743c* (*ctpJ*, G676C). Two prediction models were established using random forest and gradient boosting decision tree, we found that *Rv0197*(G344T, T2247G), *nirB* (C2037T, A2058G), *Rv0338c* (C2478T), *mrp* C649G, *ctpD* C758T, *rip* (C957T, G775T), *etgB* G1122T, *Rv3728* C2392T, and *ctpJ* G676C also contributed most to the random forest and gradient boosting decision tree (Additional file 1: Table S4, Table S9 and Additional file 2: Fig. S1). However, the *gap* SNP G654T did not contribute significantly to the gradient boosting decision tree model. Overall, our results indicated that *Rv0197* (G344T, T2247G), *nirB* (C2037T, A2058G), *Rv0338c* (C2478T), *mrp* C649G, *ctpD* C758T, *rip* (C957T, G775T), *etgB* G1122T, *Rv3728* C2392T, and *ctpJ* G676C were positively correlated with transmission clusters of *M. tuberculosis* isolates.

### Relationship between iron-related gene mutations and transmission clusters of lineages

After excluding the mutation frequency below 0.01, we identified and included a total of 40 SNPs for further analysis. In comparison to non-clustered isolates, we conducted an analysis on the relationship between 40 SNPs and clustered isolates specifically belonging to lineage 2. The GLMM revealed that five SNPs showed statistical significance for clustering (*P* < 0.05) (Table 2). Among these, two nonsynonymous SNPs and two synonymous SNPs displayed a positive correlation with clustering. These significant SNPs included *Rv0197* T2247G, *Rv1553* (f*rdB*, C87T), and *Rv2869c* (*rip*, C957T, G775T). Two prediction models were established using random forest and gradient boosting decision tree algorithms (Additional file 1: Table S5, Table S10 and Additional file 2: Fig. S2). Our findings demonstrated that *Rv0197* T2247G, *frdB* C87T, and *rip* (C957T, G775T) contributed significantly to both the random forest and gradient boosting decision tree models. Overall, our results indicated that the SNPs *Rv0197* T2247G, *frdB* C87T, and *rip* (C957T, G775T) were positively correlated with transmission clusters within *M. tuberculosis* isolates of lineage 2.

**Table 2:** Generalized linear mixed model analysis on clustered and non-clustered isolates in the lineage2 cohort

Gene	Position	SNP	Amino acid changes	P	OR (95%CI)
Rv0104	123,454	C1138T	Gln380*	0.798	0.783(0.119–5.127)
Rv0104	123,520	T1204C	Tyr402His	0.568	2.324(0.129–41.945)
Rv0197	233,016	C786T	Ala262Ala	0.969	0.98(0.367–2.618)
**Rv0197**	**234,477**	**T2247G**	Tyr749*	1.910E-10	8.339(4.341–16.021)
Rv0211	252,900	C1119T	Asp373Asp	0.232	0.494(0.155–1.572)
Rv0233	278,681	C97G	His33Asp	0.599	1.892(0.176–20.389)
Rv0252	304,923	A2058G	Lys686Lys	0.836	0.737(0.041–13.318)
Rv0252	305,188	G2323T	Val775Leu	0.807	0.762(0.086–6.776)
Rv0338c	403,980	C1862T	Ala621Val	0.043	0.011(0-0.871)
Rv0338c	404,130	A1712G	Glu571Gly	0.987	-
Rv0338c	404,326	A1516G	Arg506Gly	0.227	9.612(0.245-377.692)
Rv1175c	1,306,259	T1968C	Ala656Ala	0.871	1.197(0.137–10.478)
Rv1175c	1,307,598	G629C	Cys210Ser	0.199	0.231(0.025–2.158)
Rv1207	1,351,407	C217G	Arg73Gly	0.094	1.267(0.961–1.67)
Rv1469	1,657,249	T287C	Leu96Pro	0.213	1.243(0.883–1.749)
Rv1469	1,657,942	T980G	Val327Gly	0.989	-
**Rv1553**	**1,759,521**	**C87T**	**Leu29Leu**	2.230E-04	4.655(2.057–10.535)
Rv1937	2,189,829	C1334T	Ala445Val	0.330	1.361(0.732–2.531)
Rv1937	2,190,771	T2276C	Val759Ala	0.377	0.448(0.076–2.656)
Rv2331A	2,604,740	A1G	Met1?	0.987	-
Rv2564	2,884,068	A727C	Met243Leu	0.808	0.858(0.251–2.932)
Rv2633c	2,959,618	A203G	His68Arg	0.219	0.664(0.346–1.275)
Rv2711	3,024,021	C457A	Arg153Arg	0.096	23.477(0.572-963.141)
Rv2776c	3,084,195	C109A	Pro37Thr	0.252	1.78(0.663–4.774)
**Rv2869c**	**3,180,806**	**C957T**	**Phe319Phe**	**0.037**	6.666(1.12–39.67)
**Rv2869c**	**3,180,988**	**G775T**	**Val259Phe**	0.007	31.249(2.508-389.366)
Rv2869c	3,181,479	A284C	Lys95Thr	0.766	0.944(0.645–1.381)
Rv3224	3,600,035	A185G	Gln62Arg	0.107	1.372(0.934–2.017)
Rv3224	3,600,576	C726T	Cys242Cys	0.987	0.972(0.038–24.925)
Rv3239c	3,614,982	A2622G	Leu874Leu	0.781	1.434(0.112–18.293)
Rv3252c	3,631,457	A532C	Thr178Pro	0.880	1.048(0.567–1.939)
Rv3571	4,013,010	G594A	Leu198Leu	0.698	0.956(0.762–1.199)
Rv3674c	4,115,890	C5G	Pro2Arg	0.289	3.494(0.346–35.297)
Rv3703c	4,145,737	T1155C	Tyr385Tyr	0.379	2.869(0.274–30.026)
Rv3703c	4,146,047	C845T	Ala282Val	0.988	-
Rv3728	4,175,847	G975A	Trp325*	0.590	1.482(0.354–6.197)
Rv3728	4,176,081	C1209T	Gly403Gly	0.091	1.271(0.963–1.678)
Rv3743c	4,194,501	C873T	Phe291Phe	0.983	1.01(0.408–2.501)
Rv3743c	4,195,074	C300T	Ala100Ala	0.706	0.906(0.544–1.511)
Rv3841	4,314,645	A468G	Leu156Leu	0.110	0.031(0-2.205)

OR, odds ratio; CI, confidence interval

After excluding sites with a mutation frequency less than 0.01, we identified and included a total of 68 SNPs for further analysis. In comparison to non-clustered isolates, we conducted an analysis on the relationship between 68 SNPs and clustered isolates specifically belonging to lineage 4. The GLMM showed that 20 SNPs were found to be statistically significant for clustering (*P* < 0.05) (Additional file 1: Table S2), among which eight nonsynonymous SNPs and five synonymous SNPs were positively correlated with clustering, including *Rv0069c* (*sdaA*, A565G), *Rv0197* T2247G, *Rv0233* (*nrdB*, C97G), *Rv0338c* C2478T, *Rv1207* (*folP2*, C153A), *Rv1436* (*gap*, G654T), *Rv2711* (*ideR*, G57A), *Rv3025c* (*iscS*, C1101G), *Rv3703c* (*etgB*, G1122T, C578A), *Rv3728* C2392T, *Rv3743c* (*ctpJ*, G676C), *Rv3818* G373A. Two prediction models were established using random forest and gradient boosting decision tree (Additional file 1: Table S6, Table S11 and Additional file 2: Fig. S3). We found that *Rv0197* T2247G, *nrdB* C97G, *Rv0338c* C2478T, *folP2* C153A, *gap* G654T, *ideR* G57A, *etgB* (G1122T, C578A), *ctpJ* G676C, and *Rv3818* G373A also contributed most to the random forest and gradient boosting decision tree. Overall, our results indicated that the SNPs *Rv0197* T2247G, *nrdB* C97G, *Rv0338c* C2478T, *folP2* C153A, *gap* G654T, *ideR* G57A, *etgB* G1122T, C578A, *ctpJ* G676C, and *Rv3818* G373A were positively correlated with transmission clusters within *M. tuberculosis* isolates of lineage 4.

### Relationship between iron-related gene mutations and cross-country transmission

After excluding sites with a mutation frequency below 0.01, we identified and included a total of 90 SNPs in iron-related genes that were analyzed to assess their relationship with cross-country transmission clades. The GLMM showed that 20 SNPs were found to be statistically significant for transmission clades of cross-country (*P* < 0.05) (Table 3), among which five nonsynonymous SNPs and two synonymous SNPs were positively correlated with transmission clades, including *Rv0104* (T167G, T478G), *Rv0211* (*pckA*, A302C), *Rv0283* (*eccB3*, C423T), *Rv1436* (*gap*, G654T), *Rv1469* (*ctpD*, C758T), *Rv3703c* (*etgB*, C578A). Two prediction models were established using random forest and gradient boosting decision tree (Additional file 1: Table S7, Table S12 and Additional file 2: Fig. S4), we found that *Rv0104* (T167G, T478G), *pckA* A302C, *eccB3* C423T, *gap* G654T, *ctpD* C758T, *etgB* C578A also contributed most to the random forest and gradient boosting decision tree. Overall, our results showed that *Rv0104* (T167G, T478G), *pckA* A302C, *eccB3* C423T, *gap* G654T, *ctpD* C758T, and *etgB* C578A were positively correlated with transmission clades across different countries.

**Table 3:** Generalized linear mixed model analysis on cross-country transmission clades

Gene	Position	SNP	Amino acid changes	P	OR (95%CI)
Rv0069c	77,058	A565G	Ile189Val	0.144	2.361(0.745–7.481)
Rv0073	81,963	G288A	Leu96Leu	4.34E-07	0.145(0.069–0.307)
**Rv0104**	**122,483**	**T167G**	**Ile56Ser**	9.73E-05	3.647(1.902–6.993)
**Rv0104**	**122,794**	**T478G**	**Phe160Val**	2.830E-04	29.725(0.624-1415.389)
Rv0104	123,454	C1138T	Gln380*	1.060E-04	-
Rv0104	123,520	T1204C	Tyr402His	1.390E-04	-
Rv0104	123,745	G1429A	Gly477Arg	9.80E-05	-
Rv0197	232,574	G344T	Gly115Val	6.19E-05	-
Rv0197	233,016	C786T	Ala262Ala	1.000	-
Rv0197	233,751	A1521G	Lys507Lys	1.000	-
Rv0197	234,051	G1821A	Pro607Pro	0.999	-
Rv0197	234,477	T2247G	Tyr749*	0.977	1.009(0.535–1.903)
**Rv0211**	**252,083**	**A302C**	**Asn101Thr**	2.830E-04	7.308(2.498–21.385)
Rv0211	252,900	C1119T	Asp373Asp	1.060E-04	-
Rv0211	253,388	C1607G	Ala536Gly	1.390E-04	-
Rv0233	278,681	C97G	His33Asp	0.405	0.665(0.254–1.738)
Rv0233	278,755	C171T	Ala57Ala	0.086	2.079(0.902–4.79)
Rv0252	303,414	C549T	Phe183Phe	1.000	-
Rv0252	304,679	G1814T	Gly605Val	0.989	-
Rv0252	304,902	C2037T	Arg679Arg	1.000	-
Rv0252	304,923	A2058G	Lys686Lys	0.999	-
Rv0252	305,106	C2241T	Asp747Asp	0.999	-
Rv0252	305,188	G2323T	Val775Leu	1.000	-
Rv0283	344,288	C267G	Ser89Ser	1.000	-
**Rv0283**	**344,444**	**C423T**	**Ser141Ser**	0.038	4.349(1.085–17.428)
Rv0338c	403,364	C2478T	Pro826Pro	0.286	3.114(0.387–25.064)
Rv0338c	403,920	G1922A	Arg641His	0.043	0.089(0.008–0.93)
Rv0338c	403,980	C1862T	Ala621Val	1.060E-04	0.044(0.009–0.215)
Rv0338c	404,130	A1712G	Glu571Gly	0.999	-
Rv0338c	404,326	A1516G	Arg506Gly	0.072	0.075(0.004–1.261)
Rv0338c	405,812	A30C	Ile10Ile	0.777	0.79(0.154–4.053)
Rv1175c	1,306,259	T1968C	Ala656Ala	0.999	-
Rv1175c	1,306,615	C1612G	Pro538Ala	1.000	-
Rv1175c	1,307,598	G629C	Cys210Ser	0.442	4.826(0.087-266.177)
Rv1207	1,351,407	C217G	Arg73Gly	0.454	1.18(0.765–1.822)
Rv1229c	1,372,301	C649G	Leu217Val	0.627	1.462(0.316–6.764)
Rv1436	1,613,927	C621A	Ala207Ala	0.998	-
**Rv1436**	**1,613,960**	**G654T**	**Ala218Ala**	0.002	6.606(1.962–22.243)
Rv1469	1,657,249	T287C	Leu96Pro	0.032	0.372(0.15–0.92)
**Rv1469**	**1,657,720**	**C758T**	**Ala253Val**	0.018	4.831(1.316–17.734)
Rv1469	1,657,942	T980G	Val327Gly	0.999	-
Rv1469	1,658,312	C1350T	Ala450Ala	1.000	-
Rv1937	2,190,771	T2276C	Val759Ala	0.998	-
Rv2564	2,883,997	A656G	Gln219Arg	0.997	-
Rv2564	2,884,068	A727C	Met243Leu	0.998	-
Rv2633c	2,959,714	A107G	Asp36Gly	0.999	-
Rv2869c	3,180,806	C957T	Phe319Phe	0.998	-
Rv2869c	3,180,988	G775T	Val259Phe	0.999	-
Rv3025c	3,383,912	C1155G	Ala385Ala	0.998	-
Rv3239c	3,614,982	A2622G	Leu874Leu	0.803	1.408(0.096–20.599)
Rv3252c	3,631,160	C829T	Leu277Leu	1.000	-
Rv3571	4,012,954	G538A	Ala180Thr	0.998	-
Rv3571	4,013,010	G594A	Leu198Leu	0.197	1.316(0.867–1.995)
Rv3703c	4,145,770	G1122T	Glu374Asp	0.999	-
Rv3703c	4,146,047	C845T	Ala282Val	0.998	-
**Rv3703c**	**4,146,314**	**C578A**	**Pro193Gln**	1.390E-04	5.641(2.317–13.734)
Rv3703c	4,146,330	T562C	Leu188Leu	0.999	-
Rv3728	4,175,847	G975A	Trp325*	0.999	-
Rv3728	4,176,081	C1209T	Gly403Gly	0.005	0.238(0.087–0.65)
Rv3728	4,177,264	C2392T	Arg798Cys	0.997	-
Rv3728	4,177,280	C2408A	Ser803Tyr	1.000	-
Rv3743c	4,194,501	C873T	Phe291Phe	0.125	3.04(0.735–12.579)
Rv3743c	4,194,698	G676C	Ala226Pro	0.998	-
Rv3818	4,282,707	C259G	Pro87Ala	9.80E-05	0.125(0.044–0.355)
Rv3818	4,282,821	G373A	Ala125Thr	0.999	-
Rv3818	4,283,319	G871A	Ala291Thr	9.80E-05	0.042(0.009–0.198)
Rv3841	4,314,645	A468G	Leu156Leu	0.999	-

OR, odds ratio; CI, confidence interval

### Relationship between iron-related gene mutations and cross-regional transmission

After excluding sites with a mutation frequency below 0.01, we identified and included a total of 90 SNPs of iron-related genes. The GLMM showed that 12 SNPs were found to be statistically significant for cross-regional transmission clades (*P* < 0.05) (Additional file 1: Table S3), among which four nonsynonymous SNPs and a synonymous SNP were positively correlated with cross-regional transmission clades, including *Rv0104* T167G, *Rv0211* (*pckA*, A302C), *Rv0283* (*eccB3*, C423T), *Rv1469* (*ctpD*, C758T), *Rv3703c* (*etgB*, C578A). Two prediction models, random forest and gradient boosting decision tree, were established (Additional file 1: Table S8, Table S13 and Additional file 2: Fig. S5). The results demonstrated that *Rv0104* T167G, *pckA* A302C, *eccB3* C423T, *ctpD* C758T, and *etgB* C578A contributed significantly to both the random forest and gradient boosting decision tree models. Overall, our findings indicated that *Rv0104* T167G, *pckA* A302C, *eccB3* C423T, *ctpD* C758T, *etgB* C578A were positively correlated with transmission clades across different regions.

### Relationship between iron-related gene mutations and clade size

**Fig. 3:**
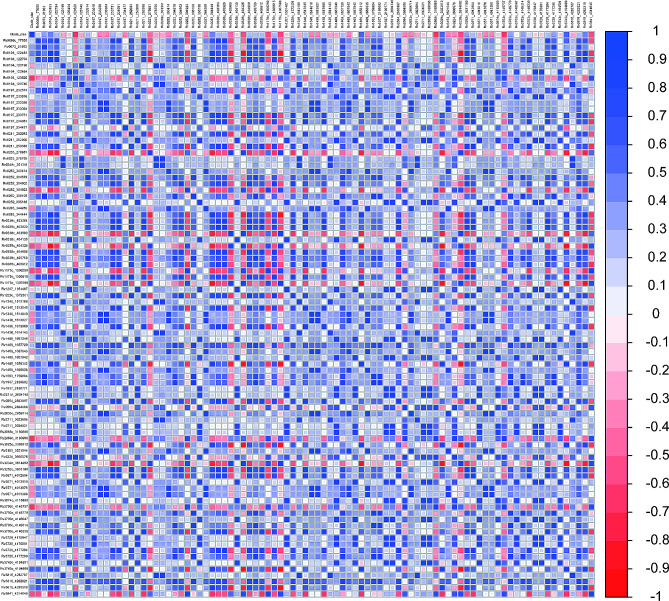
Correlation analysis of iron-related gene mutations and clade size

After excluding sites with a mutation frequency less than 0.01, we identified and included a total of 90 iron-related gene SNPs. The results showed that 78 SNPs were significantly associated with clade size (*P* < 0.05), among which 22 nonsynonymous SNPs and 11 synonymous SNPs were positively correlated with clade size, including *eccB3* C423T, *ctpD* C758T, *etgB* C578A, *rip* C957T, *etgB* G1122T, and *ctpJ* G676C. For further details refer to Fig. 3.

## Discussion

**Fig. 4:**
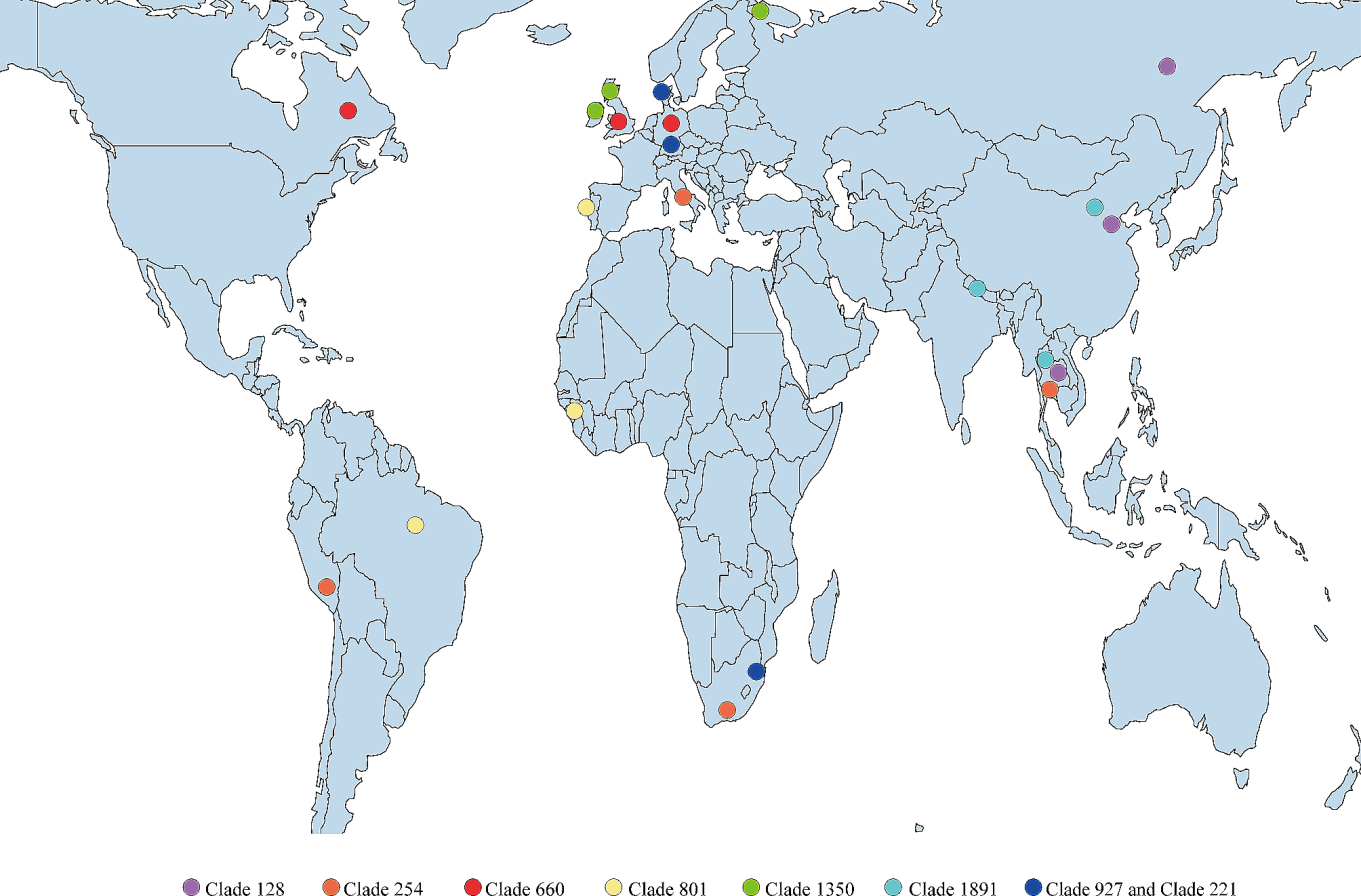
Distribution of cross-country transmission clades of *Mycobacterium tuberculosis* involves three or more countries

We have identified a relationship between iron-related gene mutations and the transmission of *M. tuberculosis* in this study. This included cluster transmission, characterized by 12 SNPs, and clade transmission, characterized by 25 SNPs. Our research findings indicated that globally, lineage 2 and lineage 4 dominate among *M. tuberculosis* isolates. Specifically, within the clusters defined by 12 SNPs, lineage 4 (*n* = 3528, 57.80%) and lineage 2 (*n* = 2131, 34.91%) were the primary contributors. Similarly, within the clades defined by 25 SNPs, lineage 4 (*n* = 4577, 54.52%) and lineage 2 (*n* = 2999, 35.72%) constitute the majority. This suggested that the transmission of *M. tuberculosis* was primarily driven by lineage 2 and lineage 4. Moreover, our findings also revealed 176 cross-country transmission clades. Among these, eight transmission clades involved three countries, while the transmission clade 254 extended across four nations: Peru, South Africa, India, and Thailand (see Fig. 4). These patterns of cross-continental transmission transcended the typical spread observed between neighboring countries. The distributional tendencies were likely intertwined with the prevalence of modern-day social activities, such as international trade, travel, and other forms of social interaction.

According to our study, two nonsynonymous SNPs of G344T and T2247G in *Rv0197* increased the risk of transmission clusters. We also noticed the SNP of T2247G in *Rv0197* was positively associated with transmission clusters of lineage2 and lineage4, which has previously been shown to be associated with enhanced transmissibility in vivo [26]. In addition, the frequent and independent occurrence in Lineage4.3/ Latin American and Mediterranean sub-lineage clonal complex (TUN4.3_CC1) of the in vivo enhanced transmission-associated mutation in *Rv0197* T2247G, could have contributed to its evolutionary success. We understand that the protein encoded by the *Rv0197* gene plays a critical role in bacterial metabolism and respiration, particularly as a putative iron oxidoreductase enzyme. We understand that the protein encoded by the *Rv0197* gene plays a critical role in bacterial metabolism and respiration, particularly as a putative iron oxidoreductase enzyme. Therefore, these SNP variations could potentially lead to structural or functional changes in the protein, thus influencing bacterial physiology [27]. Furthermore, we speculate that these SNP variations might help the bacteria adapt and survive in specific environments or hosts, possibly through alterations in host immune evasion, growth regulation, or metabolic pathways. *Rv2869c* is a mechanism of transmembrane signal transduction that functions through intramembrane proteolysis of substrates [28]. Our research revealed that the nonsynonymous SNP G775T and synonymous SNP C957T in *Rv2869c* were positively associated with transmission clusters, especially those belonging to lineage 2. Further supporting evidence from Hideki Makinoshima et al. demonstrated that *Rv2869c* played a regulatory role in cell envelope composition, in vivo growth, and in vivo persistence of *M. tuberculosis*, while also controlling multiple cell envelope-based virulence determinants [29]. Based on these collective findings, we hypothesized that these two mutations (G775T and C957T) potentially induced functional changes in the *Rv2869c* protein, impacting the formation and structure of the bacterial cell envelope, thereby influencing the transmission potential of *M. tuberculosis.* The gene *Rv0338c* encoded IspQ, a membrane-bound protein containing 2Fe-2 S and 4Fe-4 S centers, which was believed to serve as an iron-sulfur binding oxidoreductase. Given its essential role in the β-oxidation process of *M. tuberculosis*, mutations in *Rv0338c* had the potential to affect oxygen reduction reactions involved in bacterial metabolism and respiration. Further research was needed to fully comprehend the specific consequences of this synonymous mutation on the functionality of *Rv0338c* and its impact on bacterial physiology. Our study findings demonstrated a positive correlation between the synonymous SNP C2478T in *Rv0338c* and transmission clusters, particularly within lineage 4. This indicated that this specific SNP variation may have contributed to the adaptation and transmission dynamics within distinct lineages. Notably, studies had shown that mutants lacking the *etfD* gene, which interacted with *Rv0338c*, exhibited impaired growth on fatty acids or cholesterol, as well as reduced survival and growth in murine infection models [30, 31]. Our findings further underscored the significance of *Rv0338c* and its associated genes in mycobacterial physiology and pathogenesis. In our study, we also discovered that the nonsynonymous SNP G676C in *Rv3743c*, the nonsynonymous SNP G1122T in *Rv3703c*, and the nonsynonymous SNP C578A in *Rv3703c* were positively associated with transmission clusters, specifically those associated with lineage 4 isolates. *Rv3743c* is known as a cation transporter/ATPase, while *Rv3703c* is classified as an iron (II)-dependent oxidoreductase. However, the precise functional roles of these genes in the context of *M. tuberculosis* are not yet fully understood and require further investigation.

In our analysis of transmission clades, which includes cross-regional, cross-country, and clade size, we found a positive correlation between the nonsynonymous SNP C758T in *Rv1469* and cross-regional transmission, cross-country transmission, and clade size. Additionally, the nonsynonymous SNP T980G and synonymous SNP C1350T in *Rv1469* were positively associated with clade size. *Rv1469* is one of the coding genes for homologous P1B4-ATPase [32]. It belongs to the ATPase superfamily and functions as a transmembrane protein involved in the transport and regulation of metal ions. The *Rv1469* gene encodes a membrane protein annotated as the *M. tuberculosis* paralog of *Rv1469*, a member of the metal cation-transporting P1B4-ATPase subgroup. It plays an essential role in *M. tuberculosis* survival within the host. Specifically, *Rv1469* acts as a high-affinity Fe^2+^ exporter required for overcoming redox stress and adapting to the host environment [32, 33]. the nonsynonymous SNPs T167G in *Rv0104*, A302C in *Rv0211*, and C578A in *Rv3703c* were positively correlated with cross-regional transmission, cross-country transmission, and clade size. However, the specific functions of *Rv0104*, *Rv0211*, and *Rv3703c* remain unclear. It is worth noting that these associations suggest a potential link between these genetic variations and the sspread of tuberculosis across different regions and countries. However, without a clear understanding of the functions of these genes, it is difficult to determine the exact mechanisms underlying this correlation.

Additionally, our study also elucidated the association between SNPs in other iron-related genes and the transmission of *M. tuberculosis*. These genetic mutations have the potential to alter diverse physiological functions of the bacterium that are intricately linked to its transmission. By altering these iron-related pathways, SNPs in these genes may impact the fitness, virulence, or adaptive capabilities of the bacterium. This, in turn, could influence its ability to establish infections, replicate, evade host immune responses, and transmit to new hosts. Furthermore, our findings provided confirmation that both synonymous and non-synonymous mutations can impact the transmission of *M. tuberculosis*. This indicates that synonymous mutations in iron-related genes are not universally neutral, which aligns with previous studies by Xukang Shen suggesting that synonymous mutations in yeast genes are predominantly strong non-neutral mutations [34].

In this study, we have identified correlations between mutations in iron-related genes and the transmission of *M. tuberculosis*. However, it is important to acknowledge several limitations and shortcomings of our research. Firstly, although we have established these correlations, the specific impact of these mutations on the transmission dynamics of *M. tuberculosis* lacks experimental validation. Further research is needed to investigate the functional significance of these mutations and their direct influence on the transmission of the bacteria. Moreover, it is worth noting that mutations in iron-related genes may also affect other factors related to pathogenesis, such as bacterial virulence and immune response. These potential influences warrant further in-depth investigations. Understanding the broader implications of these mutations requires additional studies aimed at exploring their effects on various aspects of TB pathogenesis.

## Conclusion

The findings of this study indicate that mutations in iron-related genes could potentially elevate the risk of *M. tuberculosis* transmission, underscoring the importance of conducting additional research to explore the impact of these mutations on the control and dissemination of *M. tuberculosis*. These results offer significant insights that can inform the development of therapeutic interventions for tuberculosis.

### Electronic supplementary material

Below is the link to the electronic supplementary material.

Supplementary Material 1: Additional file 1 Table S1-S8

Supplementary Material 2: Additional file 1 Table S9-S13

Supplementary Material 3: Additional file 1 Table S14

Supplementary Material 4: Additional file 1 Table S15

Supplementary Material 5: Additional file 1 Table S16

Supplementary Material 6: Additional file 2 Fig. S1-S5

## Data Availability

The whole genome sequences have been submitted to the NCBI under the accession number PRJNA1002108.

## Abbreviations

*M. tuberculosis*: *Mycobacterium tuberculosis*
WGS: Whole-genome sequencing
SPHCC: Shandong Public Health Clinical Research Center
WRCH: Weifang Respiratory Clinical Hospital
CTAB: Cetyltrimethylammonium Bromide
QC: Quality control
SNP: Single nucleotide polymorphism
SNPs: Single nucleotide polymorphisms
NCBI: National Center for Biotechnology Information
