# Supplementary material for: Iron-related gene mutations driving global Mycobacterium tuberculosis transmission revealed by whole-genome sequencing
Source: BMC Genomics. 2024 Mar 6;25:249. doi: 10.1186/s12864-024-10152-1 (PMC10916221; doi:10.1186/s12864-024-10152-1)
Supplement: Supplementary file 1 — Supplementary Material 1: Additional file 1 Table S1-S8 [file 12864_2024_10152_MOESM1_ESM.docx]

**Table S1** Generalized linear mixed model analysis on clustered and non-clustered isolates.

| **Gene** | **Position** | **SNP** | **Amino acid changes** | ***P*** | **OR (95%CI)** |
| --- | --- | --- | --- | --- | --- |
| Rv0009 | 12555 | C88T | Leu30Leu | 0.069 | 2.219(0.939-5.245) |
| Rv0009 | 12854 | C387T | Ile129Ile | 0.990 | - |
| Rv0073 | 81963 | G288A | Leu96Leu | 0.696 | 0.572(0.035-9.42) |
| Rv0104 | 122483 | T167G | Ile56Ser | 0.011 | 0.641(0.456-0.902) |
| Rv0104 | 122794 | T478G | Phe160Val | 0.426 | 1.625(0.492-5.37) |
| Rv0104 | 123198 | T882C | Pro294Pro | 0.980 | - |
| Rv0104 | 123454 | C1138T | Gln380* | 0.990 | 1.012(0.155-6.601) |
| Rv0104 | 123520 | T1204C | Tyr402His | 0.617 | 2.131(0.11-41.252) |
| Rv0104 | 123745 | G1429A | Gly477Arg | 0.009 | 0.317(0.134-0.75) |
| Rv0197 | 232574 | G344T | Gly115Val | 1.790E-04 | 2.887(1.658-5.026) |
| Rv0197 | 233016 | C786T | Ala262Ala | 0.545 | 1.343(0.517-3.485) |
| Rv0197 | 233358 | A1128C | Val376Val | 0.996 | - |
| Rv0197 | 234051 | G1821A | Pro607Pro | 0.563 | 1.745(0.264-11.535) |
| Rv0197 | 234477 | T2247G | Tyr749* | 4.870E-14 | 3.03(2.271-4.043) |
| Rv0211 | 252083 | A302C | Asn101Thr | 1.440E-04 | 0.356(0.209-0.606) |
| Rv0211 | 252900 | C1119T | Asp373Asp | 0.002 | 0.275(0.122-0.618) |
| Rv0233 | 278681 | C97G | His33Asp | 0.107 | 1.51(0.915-2.493) |
| Rv0233 | 278755 | C171T | Ala57Ala | 0.349 | 0.017(0-88.767) |
| Rv0248c | 300193 | C611T | Ala204Val | 0.992 | - |
| Rv0249c | 301341 | G315T | Pro105Pro | 0.993 | - |
| Rv0252 | 303414 | C549T | Phe183Phe | 0.057 | 3.474(0.963-12.539) |
| Rv0252 | 304679 | G1814T | Gly605Val | 0.995 | - |
| Rv0252 | 304902 | C2037T | Arg679Arg | 1.280E-04 | 2.148(1.453-3.176) |
| Rv0252 | 304923 | A2058G | Lys686Lys | 0.015 | 10.576(1.592-70.282) |
| Rv0252 | 305106 | C2241T | Asp747Asp | 0.493 | 1.232(0.678-2.242) |
| Rv0252 | 305188 | G2323T | Val775Leu | 0.564 | 1.882(0.219-16.159) |
| Rv0265c | 317045 | C459T | Gly153Gly | 0.310 | 0.461(0.103-2.055) |
| Rv0283 | 344288 | C267G | Ser89Ser | 0.863 | 0.809(0.072-9.058) |
| Rv0283 | 344444 | C423T | Ser141Ser | 0.924 | 0.935(0.235-3.718) |
| Rv0338c | 403364 | C2478T | Pro826Pro | 3.220E-04 | 3.897(1.857-8.179) |
| Rv0338c | 403920 | G1922A | Arg641His | 1.360E-06 | 0.131(0.057-0.299) |
| Rv0338c | 403980 | C1862T | Ala621Val | 0.001 | 0.029(0.004-0.227) |
| Rv0338c | 404130 | A1712G | Glu571Gly | 0.995 | - |
| Rv0338c | 404326 | A1516G | Arg506Gly | 0.030 | 0.273(0.084-0.884) |
| Rv0338c | 405750 | A92C | Tyr31Ser | 0.995 | - |
| Rv1175c | 1306259 | T1968C | Ala656Ala | 0.304 | 2.409(0.451-12.875) |
| Rv1175c | 1307598 | G629C | Cys210Ser | 0.039 | 0.302(0.097-0.939) |
| Rv1207 | 1351407 | C217G | Arg73Gly | 0.396 | 1.118(0.864-1.446) |
| Rv1229c | 1372301 | C649G | Leu217Val | 2.520E-09 | 15.952(6.416-39.663) |
| Rv1229c | 1372362 | G588C | Lys196Asn | 0.995 | - |
| Rv1348 | 1513189 | C143T | Ala48Val | 0.987 | - |
| Rv1348 | 1514010 | G964T | Val322Phe | 0.997 | - |
| Rv1436 | 1613927 | C621A | Ala207Ala | 0.996 | - |
| Rv1436 | 1613960 | G654T | Ala218Ala | 3.190E-06 | 7.094(3.111-16.178) |
| Rv1436 | 1614143 | G837A | Leu279Leu | 0.984 | - |
| Rv1469 | 1657249 | T287C | Leu96Pro | 0.467 | 1.135(0.807-1.597) |
| Rv1469 | 1657720 | C758T | Ala253Val | 0.036 | 1.757(1.039-2.972) |
| Rv1469 | 1657843 | G881A | Arg294His | 0.991 | - |
| Rv1469 | 1657942 | T980G | Val327Gly | 0.547 | 2.136(0.181-25.186) |
| Rv1469 | 1658312 | C1350T | Ala450Ala | 0.264 | 0.331(0.047-2.303) |
| Rv1469 | 1658535 | G1573T | Val525Phe | 0.995 | - |
| Rv1553 | 1759904 | C470T | Ser157Leu | 0.594 | 2.142(0.131-35.166) |
| Rv1937 | 2190771 | T2276C | Val759Ala | 0.534 | 0.586(0.109-3.158) |
| Rv2331A | 2604740 | A1G | Met1? | 0.995 | - |
| Rv2564 | 2883997 | A656G | Gln219Arg | 0.509 | 0.635(0.165-2.443) |
| Rv2564 | 2884068 | A727C | Met243Leu | 0.569 | 1.397(0.442-4.422) |
| Rv2633c | 2959714 | A107G | Asp36Gly | 0.992 | - |
| Rv2711 | 3023684 | G120A | Thr40Thr | 0.300 | 93.886(0.018-503288.277) |
| Rv2711 | 3024021 | C457A | Arg153Arg | 0.157 | 9.055(0.428-191.758) |
| Rv2869c | 3180806 | C957T | Phe319Phe | 0.016 | 8.003(1.483-43.194) |
| Rv2869c | 3180988 | G775T | Val259Phe | 0.002 | 36.862(3.787-358.854) |
| Rv2869c | 3181479 | A284C | Lys95Thr | 0.536 | 0.889(0.613-1.29) |
| Rv3025c | 3383912 | C1155G | Ala385Ala | 0.241 | 0.481(0.141-1.637) |
| Rv3025c | 3383966 | C1101G | Val367Val | 0.239 | 0.738(0.446-1.223) |
| Rv3025c | 3384140 | C927T | Gly309Gly | 1.000 | - |
| Rv3153 | 3520977 | G471C | Leu157Leu | 0.990 | - |
| Rv3153 | 3521044 | A538G | Thr180Ala | 0.993 | - |
| Rv3224 | 3600576 | C726T | Cys242Cys | 0.539 | 2.589(0.124-53.934) |
| Rv3239c | 3614982 | A2622G | Leu874Leu | 0.279 | 1.921(0.59-6.256) |
| Rv3571 | 4013010 | G594A | Leu198Leu | 0.215 | 0.872(0.701-1.083) |
| Rv3571 | 4013076 | G660A | Gly220Gly | 0.995 | - |
| Rv3674c | 4115890 | C5G | Pro2Arg | 0.621 | 1.794(0.176-18.259) |
| Rv3703c | 4145737 | T1155C | Tyr385Tyr | 0.242 | 3.936(0.397-39.027) |
| Rv3703c | 4145770 | G1122T | Glu374Asp | <2E-16 | 62.522(25.397-153.917) |
| Rv3703c | 4146047 | C845T | Ala282Val | 0.820 | 1.325(0.118-14.928) |
| Rv3703c | 4146314 | C578A | Pro193Gln | 0.742 | 0.906(0.503-1.632) |
| Rv3703c | 4146330 | T562C | Leu188Leu | 0.334 | 0.393(0.059-2.611) |
| Rv3728 | 4175847 | G975A | Trp325* | 0.578 | 1.501(0.359-6.277) |
| Rv3728 | 4176081 | C1209T | Gly403Gly | 0.299 | 1.153(0.881-1.507) |
| Rv3728 | 4177264 | C2392T | Arg798Cys | 0.021 | 4.703(1.263-17.515) |
| Rv3728 | 4177280 | C2408A | Ser803Tyr | 0.233 | 0.663(0.338-1.302) |
| Rv3743c | 4194501 | C873T | Phe291Phe | 0.057 | 0.697(0.48-1.011) |
| Rv3743c | 4194698 | G676C | Ala226Pro | 1.000E-04 | 21.484(4.58-100.765) |
| Rv3818 | 4282707 | C259G | Pro87Ala | 0.059 | 1.846(0.977-3.49) |
| Rv3818 | 4283319 | G871A | Ala291Thr | 0.213 | 2.427(0.602-9.79) |
| Rv3841 | 4314645 | A468G | Leu156Leu | 0.117 | 0.209(0.03-1.478) |

OR, odds ratio; CI, confidence interval.

**Table S2** Generalized linear mixed model analysis on clustered and non-clustered isolates in the lineage4 cohort.

| **Gene** | **Position** | **SNP** | **Amino acid changes** | ***P*** | **OR (95%CI)** |
| --- | --- | --- | --- | --- | --- |
| Rv0009 | 12555 | C88T | Leu30Leu | 0.055 | 2.692(0.981-7.384) |
| Rv0069c | 76281 | G1342A | Gly448Ser | 0.995 | - |
| Rv0069c | 76373 | A1250G | Asp417Gly | 0.678 | 1.118(0.66-1.895) |
| Rv0069c | 76530 | C1093A | Pro365Thr | 0.997 | - |
| Rv0069c | 77058 | A565G | Ile189Val | 0.001 | 2.489(1.425-4.348) |
| Rv0073 | 81963 | G288A | Leu96Leu | 0.725 | 0.604(0.036-10.026) |
| Rv0073 | 81990 | C315A | Asn105Lys | 1.000 | - |
| Rv0104 | 122483 | T167G | Ile56Ser | 0.001 | 0.512(0.346-0.758) |
| Rv0104 | 122794 | T478G | Phe160Val | 0.51 | 1.716(0.345-8.542) |
| Rv0104 | 123475 | T1159G | Leu387Val | 0.994 | - |
| Rv0197 | 232361 | A131G | Tyr44Cys | 0.003 | 0.207(0.074-0.581) |
| Rv0197 | 232574 | G344T | Gly115Val | 0.205 | 1.456(0.815-2.603) |
| Rv0197 | 233751 | A1521G | Lys507Lys | 1.000 | - |
| Rv0197 | 234051 | G1821A | Pro607Pro | 0.503 | 1.946(0.277-13.689) |
| Rv0197 | 234477 | T2247G | Tyr749* | 1.39E-06 | 2.549(1.743-3.728) |
| Rv0211 | 252083 | A302C | Asn101Thr | 0.175 | 2.699(0.642-11.345) |
| Rv0211 | 252105 | G324A | Met108Ile | 1.000 | - |
| Rv0211 | 253388 | C1607G | Ala536Gly | 0.997 | - |
| Rv0233 | 278681 | C97G | His33Asp | 0.018 | 1.983(1.124-3.498) |
| Rv0248c | 300193 | C611T | Ala204Val | 0.994 | - |
| Rv0252 | 304679 | G1814T | Gly605Val | 0.997 | - |
| Rv0252 | 304902 | C2037T | Arg679Arg | 0.917 | 0.959(0.436-2.108) |
| Rv0252 | 304923 | A2058G | Lys686Lys | 0.997 | - |
| Rv0252 | 305106 | C2241T | Asp747Asp | 0.082 | 1.698(0.934-3.087) |
| Rv0265c | 316886 | G618A | Leu206Leu | 0.997 | - |
| Rv0265c | 317300 | C204T | Asp68Asp | 0.561 | 1.369(0.475-3.945) |
| Rv0283 | 344444 | C423T | Ser141Ser | 0.923 | 1.073(0.26-4.432) |
| Rv0338c | 403364 | C2478T | Pro826Pro | 3.740E-04 | 3.877(1.838-8.179) |
| Rv0338c | 403920 | G1922A | Arg641His | 7.81E-08 | 0.088(0.036-0.213) |
| Rv0338c | 403980 | C1862T | Ala621Val | 0.001 | 0.02(0.002-0.209) |
| Rv0338c | 404326 | A1516G | Arg506Gly | 0.036 | 0.128(0.019-0.876) |
| Rv0338c | 404809 | G1033C | Glu345Gln | 0.997 | - |
| Rv0338c | 405750 | A92C | Tyr31Ser | 0.997 | - |
| Rv0338c | 405812 | A30C | Ile10Ile | 0.096 | 2.861(0.83-9.853) |
| Rv1175c | 1306259 | T1968C | Ala656Ala | 0.635 | 2.149(0.091-50.728) |
| Rv1175c | 1306322 | G1905C | Leu635Leu | 0.995 | - |
| Rv1175c | 1306615 | C1612G | Pro538Ala | 0.253 | 1.753(0.67-4.589) |
| Rv1175c | 1307598 | G629C | Cys210Ser | 0.104 | 0.273(0.057-1.303) |
| Rv1175c | 1308161 | G66A | Arg22Arg | 0.139 | 0.285(0.054-1.505) |
| Rv1207 | 1351343 | C153A | Thr51Thr | 0.001 | 2.58(1.495-4.452) |
| Rv1229c | 1372301 | C649G | Leu217Val | 0.261 | 2.446(0.514-11.648) |
| Rv1348 | 1513845 | T799G | Ser267Ala | 0.997 | - |
| Rv1436 | 1613960 | G654T | Ala218Ala | 2.50E-06 | 9.3(3.676-23.53) |
| Rv1469 | 1657720 | C758T | Ala253Val | 0.183 | 3.607(0.544-23.912) |
| Rv1469 | 1658312 | C1350T | Ala450Ala | 0.31 | 0.366(0.053-2.545) |
| Rv1553 | 1759904 | C470T | Ser157Leu | 0.738 | 1.614(0.098-26.621) |
| Rv2564 | 2883997 | A656G | Gln219Arg | 0.82 | 0.854(0.22-3.316) |
| Rv2711 | 3023621 | G57A | Glu19Glu | 0.001 | 5.989(2.09-17.166) |
| Rv3025c | 3383912 | C1155G | Ala385Ala | 0.092 | 0.344(0.099-1.192) |
| Rv3025c | 3383966 | C1101G | Val367Val | 0.036 | 6.973(1.131-42.995) |
| Rv3153 | 3520977 | G471C | Leu157Leu | 0.995 | - |
| Rv3224 | 3599912 | T62C | Ile21Thr | 0.134 | 1.604(0.865-2.975) |
| Rv3239c | 3614770 | C2834G | Pro945Arg | 0.025 | 0.596(0.38-0.936) |
| Rv3239c | 3614982 | A2622G | Leu874Leu | 0.288 | 2.592(0.447-15.028) |
| Rv3252c | 3631160 | C829T | Leu277Leu | 0.996 | - |
| Rv3319 | 3707164 | G393T | Leu131Leu | 0.997 | - |
| Rv3571 | 4012954 | G538A | Ala180Thr | 0.165 | 0.453(0.148-1.387) |
| Rv3703c | 4145770 | G1122T | Glu374Asp | < 2e-16 | 71.522(28.491-179.543) |
| Rv3703c | 4146314 | C578A | Pro193Gln | 0.019 | 8.758(1.428-53.699) |
| Rv3703c | 4146330 | T562C | Leu188Leu | 0.373 | 0.411(0.058-2.902) |
| Rv3728 | 4177264 | C2392T | Arg798Cys | 0.007 | 6.303(1.641-24.214) |
| Rv3728 | 4177280 | C2408A | Ser803Tyr | 0.002 | 0.33(0.164-0.662) |
| Rv3743c | 4194698 | G676C | Ala226Pro | 0.001 | 13.383(2.851-62.83) |
| Rv3818 | 4282821 | G373A | Ala125Thr | 2.600E-04 | 5.918(2.279-15.369) |
| Rv3818 | 4283319 | G871A | Ala291Thr | 0.396 | 1.859(0.444-7.781) |
| Rv3841 | 4314645 | A468G | Leu156Leu | 0.997 | - |

OR, odds ratio; CI, confidence interval.

**Table S3** Generalized linear mixed model analysis on cross-regional transmission clades.

| **Gene** | **Position** | **SNP** | **Amino acid changes** | ***P*** | **OR (95%CI)** |
| --- | --- | --- | --- | --- | --- |
| Rv0069c | 77058 | A565G | Ile189Val | 0.317 | 1.828(0.561-5.95) |
| Rv0073 | 81963 | G288A | Leu96Leu | 2.29E-09 | 0.08(0.035-0.183) |
| Rv0104 | 122483 | T167G | Ile56Ser | 9.81E-05 | 3.644(1.901-6.985) |
| Rv0104 | 122794 | T478G | Phe160Val | 0.085 | 30.784(0.62-1527.397) |
| Rv0104 | 123454 | C1138T | Gln380* | 0.999 | - |
| Rv0104 | 123520 | T1204C | Tyr402His | 0.999 | - |
| Rv0104 | 123745 | G1429A | Gly477Arg | 0.996 | - |
| Rv0197 | 232574 | G344T | Gly115Val | 0.989 | - |
| Rv0197 | 233016 | C786T | Ala262Ala | 1.000 | - |
| Rv0197 | 233751 | A1521G | Lys507Lys | 1.000 | - |
| Rv0197 | 234051 | G1821A | Pro607Pro | 0.999 | - |
| Rv0197 | 234477 | T2247G | Tyr749* | 0.881 | 0.952(0.504-1.801) |
| Rv0211 | 252083 | A302C | Asn101Thr | 2.970E-04 | 7.799(2.563-23.729) |
| Rv0211 | 252900 | C1119T | Asp373Asp | 0.997 | - |
| Rv0211 | 253388 | C1607G | Ala536Gly | 0.998 | - |
| Rv0233 | 278681 | C97G | His33Asp | 0.408 | 0.666(0.255-1.742) |
| Rv0233 | 278755 | C171T | Ala57Ala | 0.087 | 2.074(0.9-4.779) |
| Rv0252 | 303414 | C549T | Phe183Phe | 1.000 | - |
| Rv0252 | 304679 | G1814T | Gly605Val | 0.989 | - |
| Rv0252 | 304902 | C2037T | Arg679Arg | 1.000 | - |
| Rv0252 | 304923 | A2058G | Lys686Lys | 0.999 | - |
| Rv0252 | 305106 | C2241T | Asp747Asp | 0.999 | - |
| Rv0252 | 305188 | G2323T | Val775Leu | 1.000 | - |
| Rv0283 | 344288 | C267G | Ser89Ser | 1.000 | - |
| Rv0283 | 344444 | C423T | Ser141Ser | 0.038 | 4.354(1.086-17.445) |
| Rv0338c | 403364 | C2478T | Pro826Pro | 0.291 | 3.08(0.382-24.839) |
| Rv0338c | 403920 | G1922A | Arg641His | 0.044 | 0.09(0.009-0.941) |
| Rv0338c | 403980 | C1862T | Ala621Val | 1.080E-04 | 0.045(0.009-0.215) |
| Rv0338c | 404130 | A1712G | Glu571Gly | 0.999 | - |
| Rv0338c | 404326 | A1516G | Arg506Gly | 0.073 | 0.075(0.004-1.273) |
| Rv0338c | 405812 | A30C | Ile10Ile | 0.777 | 0.789(0.154-4.053) |
| Rv1175c | 1306259 | T1968C | Ala656Ala | 0.999 | - |
| Rv1175c | 1306615 | C1612G | Pro538Ala | 1.000 | - |
| Rv1175c | 1307598 | G629C | Cys210Ser | 0.994 | - |
| Rv1207 | 1351407 | C217G | Arg73Gly | 0.496 | 0.844(0.519-1.375) |
| Rv1229c | 1372301 | C649G | Leu217Val | 0.993 | - |
| Rv1436 | 1613927 | C621A | Ala207Ala | 0.998 | - |
| Rv1436 | 1613960 | G654T | Ala218Ala | 0.992 | - |
| Rv1469 | 1657249 | T287C | Leu96Pro | 0.032 | 0.372(0.15-0.92) |
| Rv1469 | 1657720 | C758T | Ala253Val | 0.018 | 4.836(1.317-17.751) |
| Rv1469 | 1657942 | T980G | Val327Gly | 0.999 | - |
| Rv1469 | 1658312 | C1350T | Ala450Ala | 1.000 | - |
| Rv1937 | 2190771 | T2276C | Val759Ala | 0.998 | - |
| Rv2564 | 2883997 | A656G | Gln219Arg | 0.997 | - |
| Rv2564 | 2884068 | A727C | Met243Leu | 0.998 | - |
| Rv2633c | 2959714 | A107G | Asp36Gly | 0.999 | - |
| Rv2869c | 3180806 | C957T | Phe319Phe | 0.998 | - |
| Rv2869c | 3180988 | G775T | Val259Phe | 0.999 | - |
| Rv3025c | 3383912 | C1155G | Ala385Ala | 0.998 | - |
| Rv3239c | 3614982 | A2622G | Leu874Leu | 0.829 | 1.351(0.087-20.887) |
| Rv3252c | 3631160 | C829T | Leu277Leu | 1.000 | - |
| Rv3571 | 4012954 | G538A | Ala180Thr | 0.998 | - |
| Rv3571 | 4013010 | G594A | Leu198Leu | 0.197 | 1.315(0.867-1.995) |
| Rv3703c | 4145770 | G1122T | Glu374Asp | 0.999 | - |
| Rv3703c | 4146047 | C845T | Ala282Val | 0.998 | - |
| Rv3703c | 4146314 | C578A | Pro193Gln | 1.380E-04 | 5.641(2.317-13.734) |
| Rv3703c | 4146330 | T562C | Leu188Leu | 0.999 | - |
| Rv3728 | 4175847 | G975A | Trp325* | 0.999 | - |
| Rv3728 | 4176081 | C1209T | Gly403Gly | 0.005 | 0.238(0.087-0.65) |
| Rv3728 | 4177264 | C2392T | Arg798Cys | 0.997 | - |
| Rv3728 | 4177280 | C2408A | Ser803Tyr | 1.000 | - |
| Rv3743c | 4194501 | C873T | Phe291Phe | 0.125 | 3.043(0.735-12.594) |
| Rv3743c | 4194698 | G676C | Ala226Pro | 0.998 | - |
| Rv3818 | 4282707 | C259G | Pro87Ala | 9.88E-05 | 0.125(0.044-0.356) |
| Rv3818 | 4282821 | G373A | Ala125Thr | 0.998 | - |
| Rv3818 | 4283319 | G871A | Ala291Thr | 6.19E-05 | 0.042(0.009-0.198) |
| Rv3841 | 4314645 | A468G | Leu156Leu | 0.999 | - |

OR, odds ratio; CI, confidence interval.

**Table S4** The performance of various models for discriminating clustered isolates from non-clustered isolates.

| **Parameters** | **Training set** | | **Test set** | |
| --- | --- | --- | --- | --- |
|  | **(n=9472, 4301 clustered isolates,**  **5171 non-clustered isolates)** | | **(n=4060, 1803 clustered isolates,**  **2257 non-clustered isolates)** | |
|  | **Random Forest** | **Gradient Boosted Classification Tree** | **Random Forest** | **Gradient Boosted Classification Tree** |
| Kappa | 0.474 | 0.393 | 0.4 | 0.398 |
| AUC | 0.814 | 0.765 | 0.769 | 0.766 |
| (95% CI) | (0.806, 0.822) | (0.756, 0.774) | (0.756, 0.782) | (0.753, 0.779) |
| Sensitivity | 0.698 | 0.646 | 0.669 | 0.653 |
| (95% CI) | (0.689, 0.707) | (0.636, 0.656) | (0.655, 0.683) | (0.638, 0.668) |
| Specificity | 0.775 | 0.746 | 0.732 | 0.744 |
| (95% CI) | (0.767, 0.783) | (0.737, 0.755) | (0.718, 0.746) | (0.731, 0.757) |
| PPV | 0.721 | 0.676 | 0.666 | 0.677 |
| (95% CI) | (0.712, 0.73) | (0.667, 0.685) | (0.651, 0.681) | (0.663, 0.691) |
| NPV | 0.755 | 0.719 | 0.734 | 0.723 |
| (95% CI) | (0.746, 0.764) | (0.71, 0.728) | (0.72, 0.748) | (0.709, 0.737) |
| PLR | 2.943 | 2.408 | 2.506 | 2.442 |
| (95% CI) | (2.928, 2.958) | (2.392, 2.424) | (2.484, 2.528) | (2.418, 2.466) |
| NIR | 0.340 | 0.415 | 0.399 | 0.409 |
| (95% CI) | (0.306, 0.374) | (0.384, 0.446) | (0.352, 0.446) | (0.361, 0.457) |
| Accuracy | 0.740 | 0.701 | 0.704 | 0.703 |
| (95% CI) | (0.731, 0.749) | (0.692, 0.71) | (0.69, 0.718) | (0.689, 0.717) |

AUC, area under the curve; PPV, positive predictive value; NPV, negative predictive value; PLR, positive likelihood ratio; NLR, negative likelihood ratio; CI, confidence.

**Table S5** The performance of various models for discriminating clustered isolates from non-clustered isolates in lineage2 cohort.

| **Parameters** | **Training set** | | **Test set** | |
| --- | --- | --- | --- | --- |
|  | **(n=3594, 1482 clustered isolates,** | | **(n=1541, 649 clustered isolates,** | |
|  | **2112 non-clustered isolates)** | | **892 non-clustered isolates)** | |
|  | **Random Forest** | **Gradient Boosted Classification Tree** | **Random Forest** | **Gradient Boosted Classification Tree** |
| Kappa | 0.415 | 0.416 | 0.454 | 0.389 |
| AUC | 0.764 | 0.756 | 0.76 | 0.721 |
| (95% CI) | (0.75, 0.778) | (0.742, 0.77) | (0.739, 0.781) | (0.699, 0.743) |
| Sensitivity | 0.538 | 0.54 | 0.575 | 0.502 |
| (95% CI) | (0.522, 0.554) | (0.524, 0.556) | (0.55, 0.6) | (0.477, 0.527) |
| Specificity | 0.861 | 0.862 | 0.864 | 0.868 |
| (95% CI) | (0.85, 0.872) | (0.851, 0.873) | (0.847, 0.881) | (0.851, 0.885) |
| PPV | 0.731 | 0.741 | 0.755 | 0.713 |
| (95% CI) | (0.717, 0.745) | (0.727, 0.755) | (0.734, 0.776) | (0.69, 0.736) |
| NPV | 0.726 | 0.718 | 0.736 | 0.727 |
| (95% CI) | (0.711, 0.741) | (0.703, 0.733) | (0.714, 0.758) | (0.705, 0.749) |
| PLR | 2.673 | 2.632 | 2.864 | 2.609 |
| (95% CI) | (2.646, 2.7) | (2.604, 2.66) | (2.824, 2.904) | (2.569, 2.649) |
| NIR | 0.374 | 0.38 | 0.349 | 0.383 |
| (95% CI) | (0.317, 0.431) | (0.322, 0.438) | (0.258, 0.44) | (0.3, 0.466) |
| Accuracy | 0.728 | 0.725 | 0.742 | 0.723 |
| (95% CI) | (0.713, 0.743) | (0.71, 0.74) | (0.72, 0.764) | (0.701, 0.745) |

AUC, area under the curve; PPV, positive predictive value; NPV, negative predictive value; PLR, positive likelihood ratio; NLR, negative likelihood ratio; CI, confidence.

**Table S6** The performance of various models for discriminating clustered isolates from non-clustered isolates in lineage4 cohort.

| **Parameters** | **Training set** | | **Test set** | |
| --- | --- | --- | --- | --- |
|  | **(n=4549, 2434 clustered isolates,**  **2115 non-clustered isolates)** | | **(n=1950, 1094 clustered isolates,**  **856 non-clustered isolates)** | |
|  | **Random Forest** | **Gradient Boosted Classification Tree** | **Random Forest** | **Gradient Boosted Classification Tree** |
| Kappa | 0.458 | 0.357 | 0.381 | 0.349 |
| AUC | 0.817 | 0.775 | 0.773 | 0.754 |
| (95% CI) | (0.806, 0.828) | (0.763, 0.787) | (0.754, 0.792) | (0.735, 0.773) |
| Sensitivity | 0.819 | 0.828 | 0.793 | 0.827 |
| (95% CI) | (0.808, 0.83) | (0.817, 0.839) | (0.775, 0.811) | (0.81, 0.844) |
| Specificity | 0.635 | 0.521 | 0.582 | 0.516 |
| (95% CI) | (0.621, 0.649) | (0.506, 0.536) | (0.56, 0.604) | (0.494, 0.538) |
| PPV | 0.721 | 0.676 | 0.708 | 0.661 |
| (95% CI) | (0.708, 0.734) | (0.662, 0.69) | (0.688, 0.728) | (0.64, 0.682) |
| NPV | 0.753 | 0.715 | 0.687 | 0.724 |
| (95% CI) | (0.74, 0.766) | (0.702, 0.728) | (0.666, 0.708) | (0.704, 0.744) |
| PLR | 2.919 | 2.37 | 2.26 | 2.389 |
| (95% CI) | (2.898, 2.94) | (2.347, 2.393) | (2.22, 2.3) | (2.356, 2.422) |
| NIR | 0.343 | 0.422 | 0.442 | 0.419 |
| (95% CI) | (0.294, 0.392) | (0.377, 0.467) | (0.369, 0.515) | (0.352, 0.486) |
| Accuracy | 0.733 | 0.689 | 0.700 | 0.682 |
| (95% CI) | (0.72, 0.746) | (0.676, 0.702) | (0.68, 0.72) | (0.661, 0.703) |

AUC, area under the curve; PPV, positive predictive value; NPV, negative predictive value; PLR, positive likelihood ratio; NLR, negative likelihood ratio; CI, confidence.

**Table S7** The performance of various models for discriminating cross-country from non-cross-country transmission clades.

| **Parameters** | **Training set** | | **Test set** | |
| --- | --- | --- | --- | --- |
|  | **(n=5876, 505 cross-country isolates,**  **5371 non-cross-country isolates)** | | **(n=2519,215 cross-country isolates,**  **2304 non-cross-country isolates)** | |
|  | **Random Forest** | **Gradient Boosted Classification Tree** | **Random Forest** | **Gradient Boosted Classification Tree** |
| Kappa | 0.039 | 0.047 | 0.024 | 0.015 |
| AUC | 0.761 | 0.757 | 0.732 | 0.722 |
| (95% CI) | (0.75, 0.772) | (0.746, 0.768) | (0.715, 0.749) | (0.705, 0.739) |
| Sensitivity | 0.022 | 0.026 | 0.014 | 0.009 |
| (95% CI) | (0.018, 0.026) | (0.022, 0.03) | (0.009, 0.019) | (0.005, 0.013) |
| Specificity | 1.000 | 1.000 | 1.000 | 1.000 |
| (95% CI) | (1.000, 1.000) | (1.000, 1.000) | (1.000, 1.000) | (1.000, 1.000) |
| PPV | 0.917 | 0.929 | 0.750 | 0.667 |
| (95% CI) | (0.910, 0.924) | (0.922, 0.936) | (0.733, 0.767) | (0.649, 0.685) |
| NPV | 0.916 | 0.918 | 0.914 | 0.909 |
| (95% CI) | (0.909, 0.923) | (0.911, 0.925) | (0.903, 0.925) | (0.898, 0.920) |
| PLR | 10.881 | 11.340 | 8.733 | 7.325 |
| (95% CI) | (10.870, 10.892) | (11.329, 11.351) | (8.72, 8.746) | (7.313, 7.337) |
| NIR | 0.092 | 0.088 | 0.115 | 0.137 |
| (95% CI) | (0.007, 0.177) | (-0.004, 0.180) | (0.045, 0.185) | (0.078, 0.196) |
| Accuracy | 0.916 | 0.918 | 0.914 | 0.909 |
| (95% CI) | (0.909, 0.923) | (0.911, 0.925) | (0.903, 0.925) | (0.898, 0.920) |

AUC, area under the curve; PPV, positive predictive value; NPV, negative predictive value; PLR, positive likelihood ratio; NLR, negative likelihood ratio; CI, confidence.

**Table S8** The performance of various models for discriminating cross-regional from non-cross-regional transmission clades.

| **Parameters** | **Training set** | | **Test set** | |
| --- | --- | --- | --- | --- |
|  | **(n=5876, 5385 non-cross-regional isolates,491 cross-regional isolates)** | | **(n=2519, 210 cross-regional isolates,**  **2309 non-cross-regional isolates)** | |
|  | **Random Forest** | **Gradient Boosted Classification Tree** | **Random Forest** | **Gradient Boosted Classification Tree** |
| Kappa | 0.053 | 0.126 | 0.046 | 0.101 |
| AUC | 0.757 | 0.769 | 0.773 | 0.759 |
| (95% CI) | (0.746, 0.768) | (0.758, 0.78) | (0.757, 0.789) | (0.742, 0.776) |
| Sensitivity | 0.031 | 0.084 | 0.029 | 0.066 |
| (95% CI) | (0.027, 0.035) | (0.077, 0.091) | (0.022, 0.036) | (0.056, 0.076) |
| Specificity | 0.999 | 0.994 | 0.998 | 0.995 |
| (95% CI) | (0.998, 1.0) | (0.992, 0.996) | (0.996, 1.0) | (0.992, 0.998) |
| PPV | 0.833 | 0.598 | 0.545 | 0.577 |
| (95% CI) | (0.823, 0.843) | (0.585, 0.611) | (0.526, 0.564) | (0.558, 0.596) |
| NPV | 0.919 | 0.908 | 0.919 | 0.915 |
| (95% CI) | (0.912, 0.926) | (0.901, 0.915) | (0.908, 0.93) | (0.904, 0.926) |
| PLR | 10.256 | 6.496 | 6.706 | 6.752 |
| (95% CI) | (10.247, 10.265) | (6.489, 6.503) | (6.696, 6.716) | (6.741, 6.763) |
| NIR | 0.098 | 0.154 | 0.149 | 0.148 |
| (95% CI) | (0.04, 0.156) | (0.12, 0.188) | (0.102, 0.196) | (0.098, 0.198) |
| Accuracy | 0.918 | 0.904 | 0.917 | 0.911 |
| (95% CI) | (0.911, 0.925) | (0.896, 0.912) | (0.906, 0.928) | (0.9, 0.922) |

AUC, area under the curve; PPV, positive predictive value; NPV, negative predictive value; PLR, positive likelihood ratio; NLR, negative likelihood ratio; CI, confidence.
