# Supplementary material for: Iron-related gene mutations driving global Mycobacterium tuberculosis transmission revealed by whole-genome sequencing
Source: BMC Genomics. 2024 Mar 6;25:249. doi: 10.1186/s12864-024-10152-1 (PMC10916221; doi:10.1186/s12864-024-10152-1)
Supplement: Supplementary file 6 — Supplementary Material 6: Additional file 2 Fig. S1-S5 [file 12864_2024_10152_MOESM6_ESM.docx]

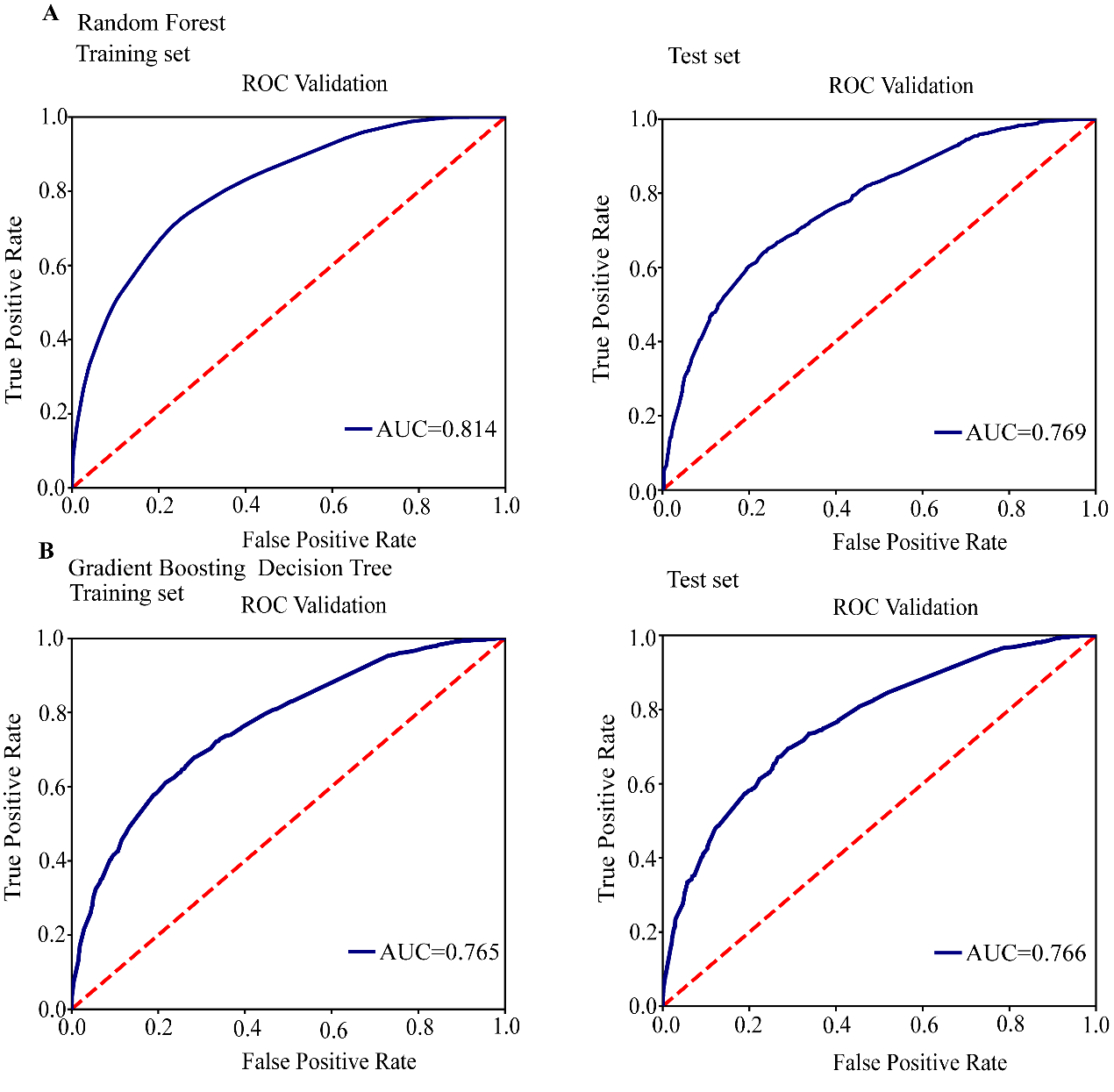


**Additional file 2: Fig. S1** ROC curve analysis was conducted to evaluate the performance of models for cluster analysis. (A) ROC analysis showing the performance of the random forest model. (B) ROC analysis showing the performance of the gradient boosting decision tree.


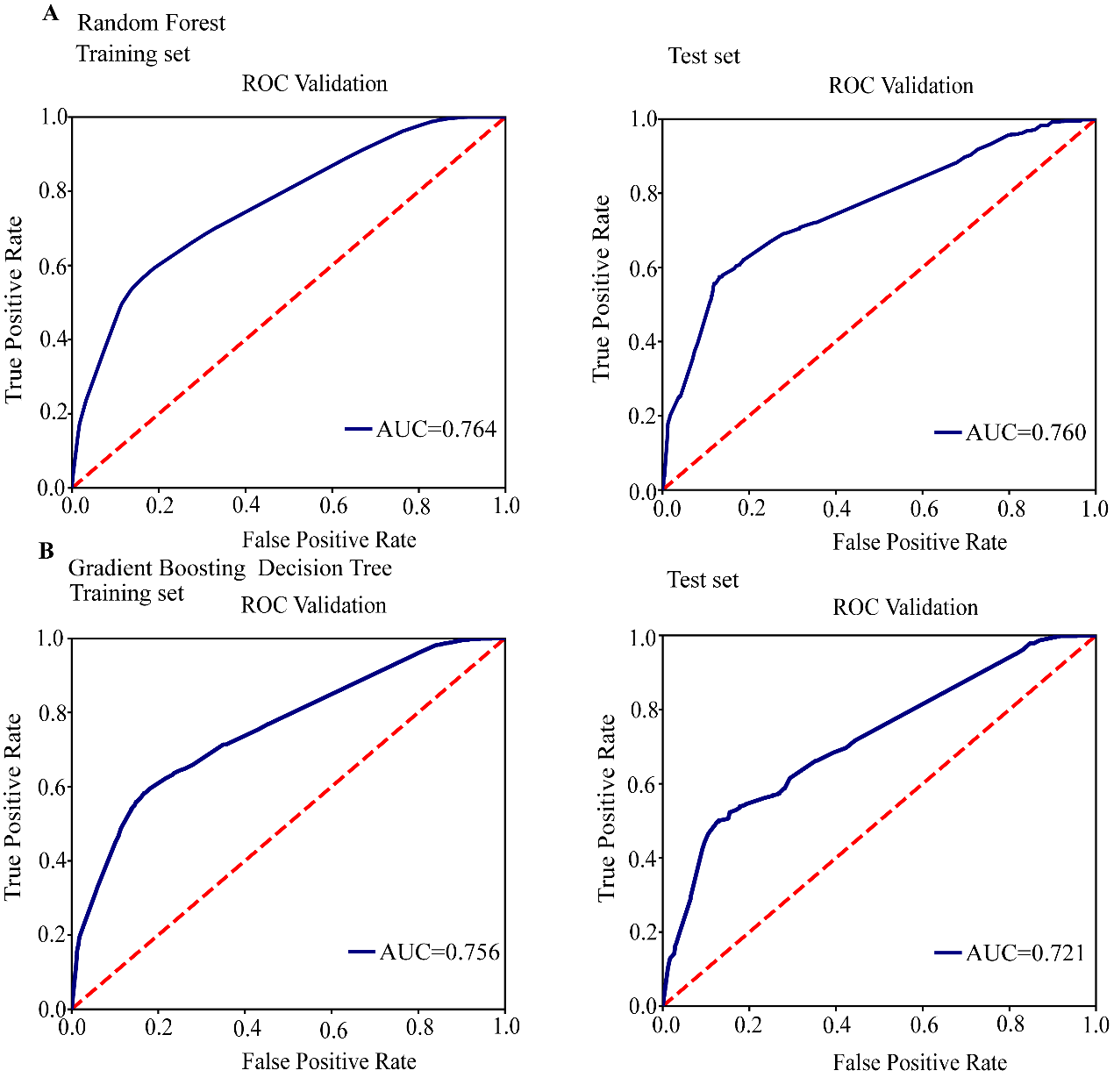


**Additional file 2: Fig. S2** ROC curve analysis was conducted to evaluate the performance of models for cluster analysis within lineage 2. (A) ROC analysis showing the performance of the random forest model. (B) ROC analysis showing the performance of the gradient boosting decision tree.


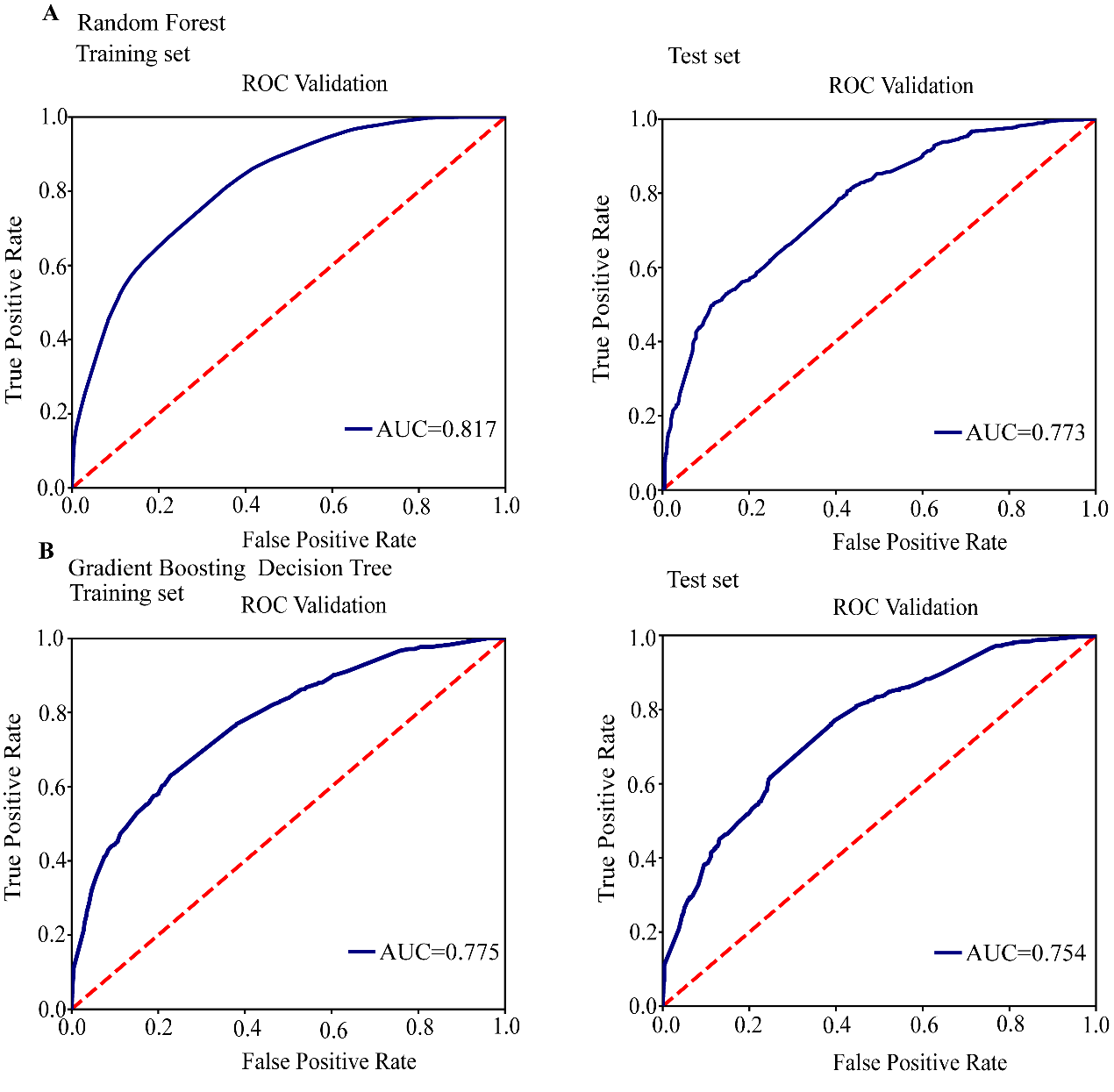


**Additional file 2: Fig. S3** ROC curve analysis was conducted to evaluate the performance of models for cluster analysis within lineage 4. (A) ROC analysis showing the performance of the random forest model. (B) ROC analysis showing the performance of the gradient boosting decision tree.


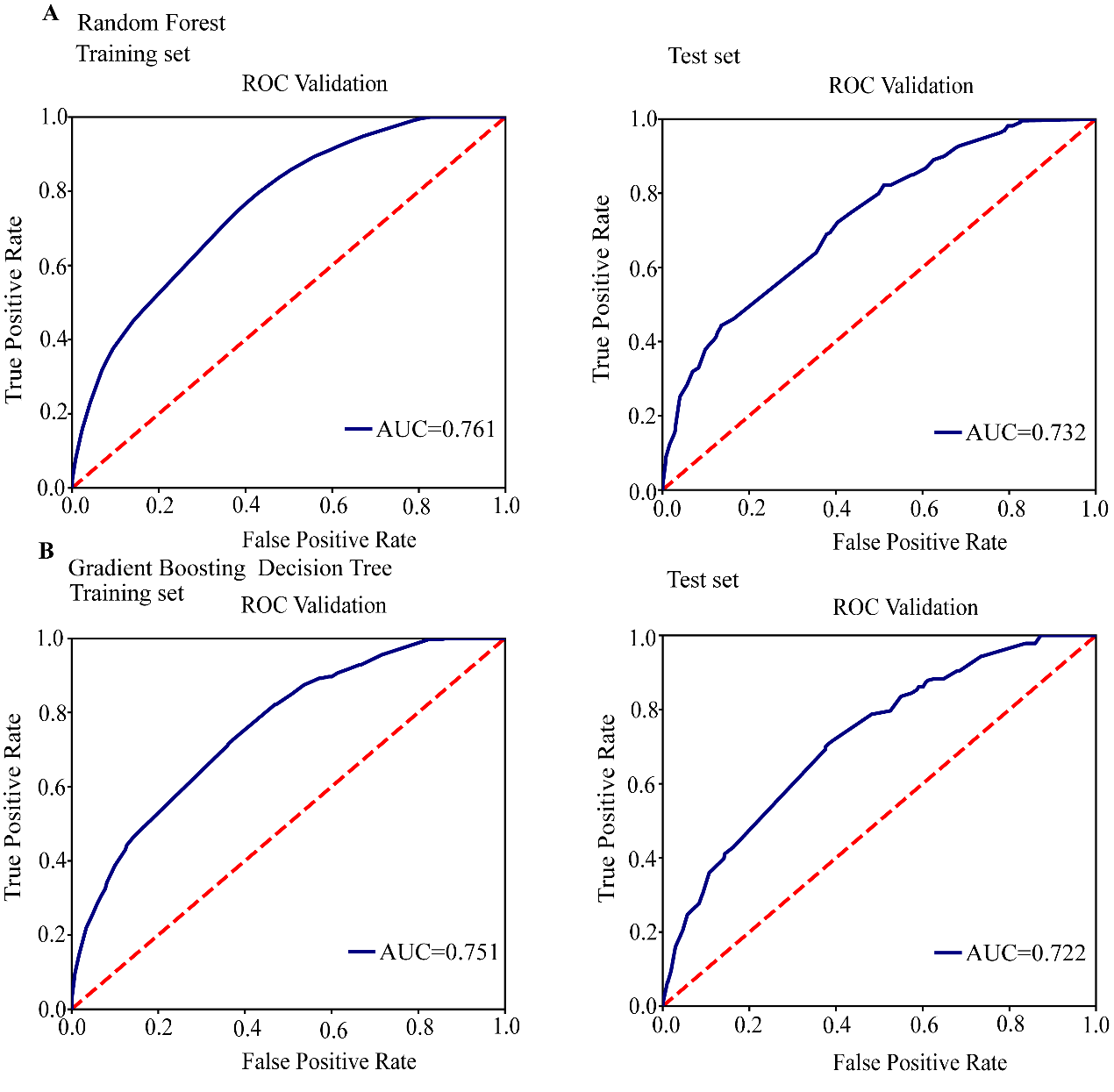


**Additional file 2: Fig. S4** The ROC curve analysis was conducted to evaluate the performance of models for cross-country transmission clades analysis. (A) ROC analysis showing the performance of the random forest model. (B) ROC analysis showing the performance of the gradient boosting decision tree.


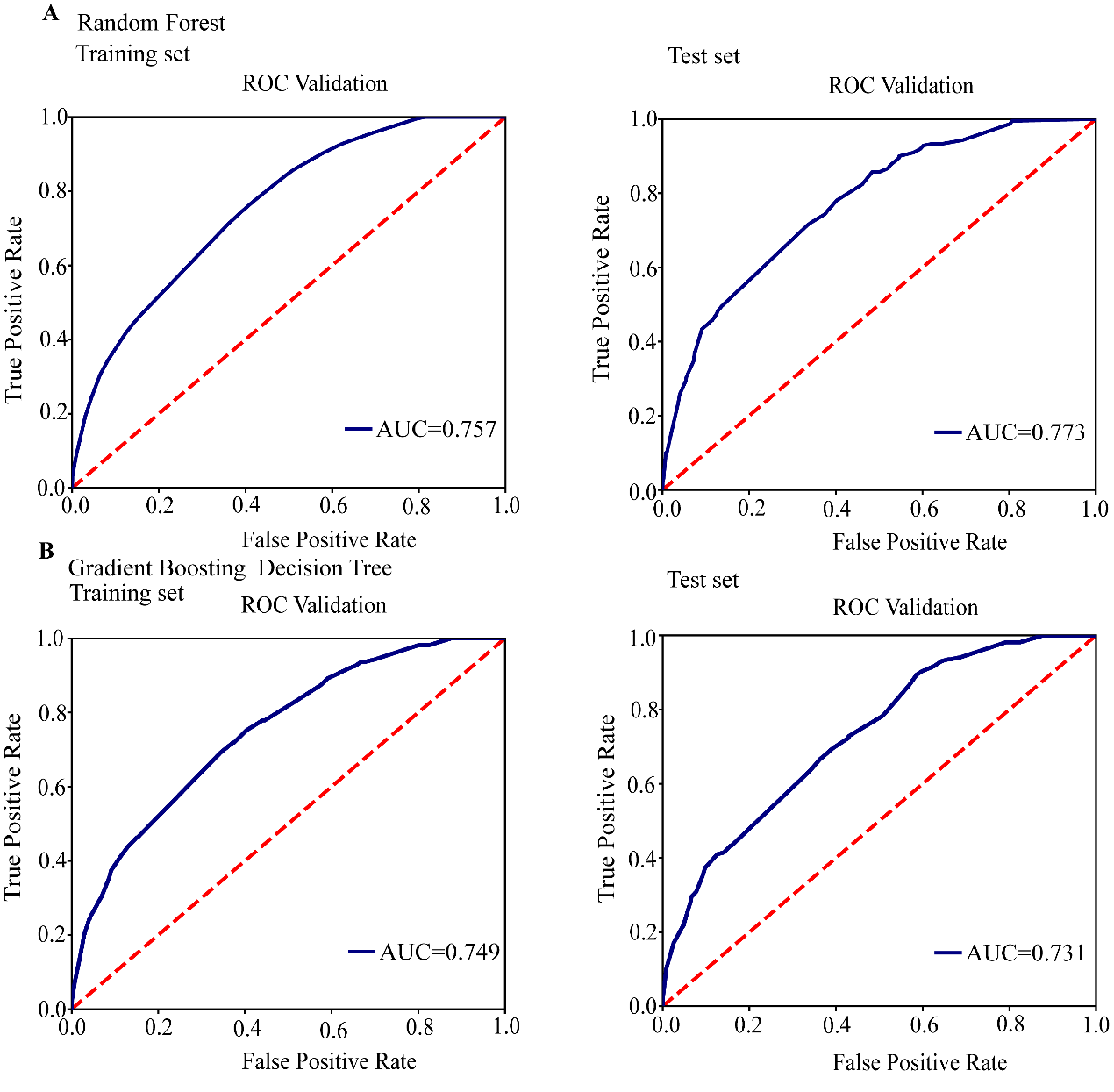


**Additional file 2: Fig. S5** The ROC curve analysis was conducted to evaluate the performance of models for cross-regional transmission clades analysis. (A) ROC analysis showing the performance of the random forest model. (B) ROC analysis showing the performance of the gradient boosting decision tree.
